# Identification of miRNA-mRNA associations in hepatocellular carcinoma using hierarchical integrative model

**DOI:** 10.1186/s12920-020-0706-1

**Published:** 2020-03-30

**Authors:** Rency S. Varghese, Yuan Zhou, Megan Barefoot, Yifan Chen, Cristina Di Poto, Abdalla Kara Balla, Everett Oliver, Zaki A. Sherif, Deepak Kumar, Alexander H. Kroemer, Mahlet G. Tadesse, Habtom W. Ressom

**Affiliations:** 1 Department of Oncology, Lombardi Comprehensive Cancer Center, Georgetown University, Room 175, Building D, 4000 Reservoir Rd NW, Washington, DC, 20057 USA; 2 MedStar Georgetown University Hospital, Washington, DC, USA; 3 Department of Biochemistry & Molecular Biology, College of Medicine, Howard University, Washington DC, USA; 4 Department of Pharmaceutical Sciences, North Carolina Central University, Durham, NC USA; 5 Department of Mathematics and Statistics, Georgetown University, Washington DC, USA

**Keywords:** miRNA, mRNA, Hierarchical integrative model, Pathway analysis, hepatocellular carcinoma, Next generation sequencing

## Abstract

**Background:**

The established role miRNA-mRNA regulation of gene expression has in oncogenesis highlights the importance of integrating miRNA with downstream mRNA targets. These findings call for investigations aimed at identifying disease-associated miRNA-mRNA pairs. Hierarchical integrative models (HIM) offer the opportunity to uncover the relationships between disease and the levels of different molecules measured in multiple omic studies.

**Methods:**

The HIM model we formulated for analysis of mRNA-seq and miRNA-seq data can be specified with two levels: (1) a mechanistic submodel relating mRNAs to miRNAs, and (2) a clinical submodel relating disease status to mRNA and miRNA, while accounting for the mechanistic relationships in the first level.

**Results:**

mRNA-seq and miRNA-seq data were acquired by analysis of tumor and normal liver tissues from 30 patients with hepatocellular carcinoma (HCC). We analyzed the data using HIM and identified 157 significant miRNA-mRNA pairs in HCC. The majority of these molecules have already been independently identified as being either diagnostic, prognostic, or therapeutic biomarker candidates for HCC. These pairs appear to be involved in processes contributing to the pathogenesis of HCC involving inflammation, regulation of cell cycle, apoptosis, and metabolism. For further evaluation of our method, we analyzed miRNA-seq and mRNA-seq data from TCGA network. While some of the miRNA-mRNA pairs we identified by analyzing both our and TCGA data are previously reported in the literature and overlap in regulation and function, new pairs have been identified that may contribute to the discovery of novel targets.

**Conclusion:**

The results strongly support the hypothesis that miRNAs are important regulators of mRNAs in HCC. Furthermore, these results emphasize the biological relevance of studying miRNA-mRNA pairs.

## Background

MicroRNAs (miRNAs) play vital roles in many biological processes, including differentiation, cell signaling, and response to infection. Overwhelming evidence indicates that dysregulation of miRNA expression is a cause or indicator of several disease processes, including many Cancer*s. Mi*croRNA dysregulation has been linked to cancer initiation and progression where miRNAs act as tumor suppressors or oncogenes, regulating multiple pathways including cell proliferation, differentiation, apoptosis, metastasis and angiogenesis [1]. Recent studies have highlighted several miRNAs that are differentially expressed in cancer stem cells establishing the role of miRNAs in targeting genes and pathways supporting cancer stemness [2]. Several miRNAs and their targets are involved in known risks and hallmarks of liver diseases and HCC, such as steatosis, fibrosis, and cirrhosis [3]. For example, a series of experiments conducted in hepatoma cells have found miR-93-5p to be upregulated and act as a promoter of cell proliferation. Furthermore, the inverse correlation between miR-93-5p and PPARGC1A gene expression was validated in the TCGA liver cancer data [4]. Mature miRNAs are highly stable and have great utility as biomarkers of diagnosis/prognosis and disease progression.

The importance of miRNA in carcinogenesis can be better understood through linking miRNA with respective target genes. The impact that miRNA-mRNA pairs have in carcinogenesis also highlights the potential of miRNAs to become therapeutic targets [5]. Many studies demonstrate that disruption of miRNA-mRNA paired regulation contributes to tumorigenesis. For example, a study consisting of African American participants suggests that miRNA-mRNA pairs play a critical role in the activation of oncogenic pathways in prostate cancer [6]. A co-expression-based network approach has identified clustered regulatory circuits of miRNA-mRNA modules in hepatocyte, inflammatory-stress and proliferative process-activated subcategories of HCC [7]. Thus, the associations between miRNAs and their target genes, i.e., up or down regulated genes, is currently an area of significant research interest.

MicroRNAs either suppress an mRNA through translational repression or accelerated degradation, or activate an mRNA through stimulated stabilization or stimulated translation. Oncogenic miRNAs (oncomiRs) act directly on mRNAs from genes with pro-apoptotic or anti-proliferative roles [8]. Conversely, mRNAs either suppress miRNA through accelerated degradation, or activate miRNA by sequestering it from degradation. Typically, miRNA-mRNA associations exist as reciprocal pairs whereby cells select either high/low level of mRNA/miRNA or the opposite expression pattern [9]. However, dual-upregulation of paired miRNA-mRNA has been experimentally validated as an existing relationship observed in cancer [10]. Also, the action of miRNAs on their mRNA targets is difficult to characterize, because each miRNA has multiple mRNA targets and vice versa. The correct identification of an interaction is still a challenge and are typically addressed using prediction methods followed by experimental validation of these miRNA-mRNA interactions. As miRNA-mRNA regulation generally exists in various biological systems, understanding the functionality of miRNA-mRNA association is important for learning its underlying design principle.

Specific miRNA-mRNA interactions and high-throughput analyses of the downstream effects of miRNAs on mRNAs have been the focus of many research studies [11, 12]. In typical experiments, miRNA target prediction tools like TargetScan and MiRanda, are utilized to explore the targeted mRNAs that are regulated by differentially expressed miRNAs. Then genes that closely correlate with the expression profiles with activation and inhibition are selected [13]. A recent study used the Pearson correlation coefficient between 84 key differentially expressed genes and differentially expressed miRNAs and long non-coding RNAs (lncRNA). The relationship of miRNA–lncRNA, miRNA–mRNA, and mRNA–lncRNA with correlation coefficient greater than 0.5 was used to select the pair of molecules [14].

Hierarchical integrative models (HIMs) offer the opportunity to uncover the relationships between disease and the levels of different molecules measured in multiple omic studies. In the past, they have been successfully implemented for integrating DNA methylation, copy number variation (CNV), and gene expression data in relation with survival outcomes [15]. Specifically, modeling approaches based on penalized likelihood methods and expectation-maximization algorithms were applied to various biological relationship scenarios between different molecular features to determine their effects on clinical outcome. In this paper, we formulated a HIM adapted for application to identify miRNA-mRNA associations related to HCC. We accomplished this through analysis of mRNA-seq and miRNA-seq data generated in house from HCC patients paired tumor and normal liver tissues. We demonstrate that our model offers insights into the molecular mechanisms of HCC. Through pathway and network analysis of selected miRNA-mRNA associations we uncover the biological relevance of these pairs in HCC.

## Methods

### Characteristics of participants

In this study, liver tissues from 30 adult patients recruited at Medstar Georgetown University Hospital (MGUH), Washington, DC were used. Table 1 presents characteristics of these patients, who are a subset of 65 participants (40 HCC cases and 25 patients with liver cirrhosis) whose liver tissues samples were analyzed by mRNA-seq and miRNA-seq. In this study, we focused on mRNA-seq and miRNA-seq data (GU dataset) obtained by analysis of tumor and adjacent normal tissues from 30 HCC cases. The patients were diagnosed to have HCC based on well-established diagnostic imaging criteria and/or histology. Clinical stages for HCC cases were determined based on the tumor-node-metastasis (TNM) staging system. Tissues were donated by patients scheduled for elective surgical procedures due to the cancerous or potentially cancerous conditions and/or other related benign conditions. These provide a source of both tumor and normal-control tissues. Prior to enrollment in the study, the patients were consented to participate using IRB-approved informed consent and HIPAA authorization forms.

**Table 1:** Characteristics of subjects whose liver tissues are analyzed by mRNA-seq and miRNA-seq in GU dataset

Characteristics			HCC (^**a**^***N = 30***)
**Age**	*Mean (SD)*		61.67 (12.2)
**Gender**	*Male*		83.33%
**Race**	*AA*		30%
	*EA*		37%
	*Asian*		33%
**BMI**	*Mean (SD)*		26.17 (5.4)
**Smoking**	*Yes*		66.67%
	*No*		33.33%
**Alcohol**	*Yes*		47%
	*No*		53%
**HCV Serology**	*HCV Ab+*	*Reactive*	37%
	*HCV Ab+*	*Non-Reactive*	60%
	*HCV Ab+*	*Unavailable*	3.33%
	*HCV RNA+*	*Reactive*	16.67%
	*HCV RNA+*	*Non-Reactive*	17%
	*HCV RNA+*	*Unavailable*	63%
**HBV Serology**	*anti HBC+*	*Reactive*	10%
	*anti HBC+*	*Non-Reactive*	3.33%
	*anti HBC+*	*Unavailable*	87%
	*HBs Ag+*	*Reactive*	17%
	*HBs Ag+*	*Non-Reactive*	70%
	*HBs Ag+*	*Unavailable*	13%
**MELD**	*Mean (SD)*		10.04 (4.5)
	*MELD < 10*		56.67%
**AFP** ***(ng/mL)***	*Median (IQR)*		4.4 (14.05)
**Child Pugh Score**	*Mean (SD)*		6.14 (1.5)
	*Median (IQR)*		6 (1)
**Child Pugh Class**	*A*		73%
	*B*		13.33%
	*C*		3.33%
**AST** ***(IU/L)***	*Median (IQR)*		123 (158)
**ALT** ***(IU/L)***	*Median (IQR)*		117 (152)
**HCC Stage** ***(TNM Staging)***	*Stage I*		43%
	*Stage II*		24%
	*Stage III & IV*		33%

^a^Adjacent normal tissue for the HCC patients are available for this study

Furthermore, we analyzed miRNA-seq and mRNA-seq data obtained from the TCGA Research Network (TCGA dataset). The dataset we used in this study is a small subset of the TCGA data obtained by analysis of tumor and adjacent normal liver tissues from 49 patients (Table 2). These patients were matched using the TCGA patient identifiers to select their corresponding normal tissues.

**Table 2:** Characteristics of subjects from TCGA-LIHC cohort

Characteristics		HCC (^**a**^***N = 49***)
**Age**	*Mean (SD)*	60.77 (16.2)
**Gender**	*Male*	55.10%
**Race**	*AA*	14%
	*EA*	67%
	*Asian*	12%
	*not report*	6.12%
**HCC Stage**	*Stage I*	34.69%
	*Stage II*	22.45%
	*Stage III & IV*	26.53%
	*Unknown*	16.33%

^a^Adjacent normal tissue for 49 HCC patients are available for this study

### RNA isolation and quality assessment

Total RNA was isolated from 20 mg frozen liver tissue using the Zymo Direct-zol RNA Miniprep Plus Kit (Zymo Research) according to the manufacturer’s instructions. The RNA quality and quantity were analyzed with UV-VIS using the NanoDrop spectrophotometer (Thermo Fisher), and fluorometry using the Qubit 2.0 Fluorometer (Thermo Fisher). RNA integrity was assessed using the Agilent RNA 6000 Nano Kit on the Agilent 2100 Bioanalyzer. This work was performed in the Genomics and Epigenomics Shared Resource (GESR) at GUMC.

### mRNA expression profiling

RNA samples extracted from liver tissues were analyzed by mRNA-seq. Briefly, indexed paired-end sequencing libraries were prepared from 10 ng total RNA using the TruSeq RNA Access Library Prep Kit (Illumina). Paired-end sequencing was performed on the HiSeq 4000 System (Illumina) using 150 bp paired-end (PE150) read mode.

The mRNA-seq data contained an average of 33 M reads per sample. The raw data recorded in FASTQ files were imported into Partek® Flow® software, (Partek Inc., St. Louis, MO, USA) for quality assessment and mRNA-seq data analysis. Quality assessment was performed at each step of the procedure including RNA isolation, library preparation and sequencing. Reads containing the adapters or low quality (Qscore <=5) bases were removed prior to alignment. For the mRNA-seq data, the average Q30 (base count of Phred value > 30/ total base count) was 93.99% and the average GC content was 52.1%.

Alignment to the hg38 reference genome was performed using the spliced transcripts alignment to a reference (STAR) algorithm, with 89.6% alignment. The aligned reads were then filtered to remove duplicate reads and then quantified to the transcriptome through an Expectation Maximization (EM) method. To compare the gene expression between two conditions and eliminate systematic effects that are not associated with the biological differences of interest, we needed to normalize the samples. TMM (Trimmed mean of M-values) method was applied to estimate appropriate scaling factors for normalization. The normalized count data were exported from the Partek Flow software for further data analysis. After conducting the TMM normalization, Principal Components Analysis was used for outlier screening. One Adj-N tissue sample was detected as an outlier. Therefore, we deleted this Adj-N tissue sample and its corresponding HCC tissue sample from the further analysis.

During the sample preparation of the mRNA-seq experiment, four samples were randomly selected and were included as blinded samples for subsequent quality assessment. During data acquisition, these samples were marked as unknowns. Using cluster analysis of the result mRNA-seq data, we successfully clustered the ‘Unknown’ samples into their correct groups. This confirms the quality of the mRNA-seq data we acquired and preprocessed, prior to analyzing the data using hierarchical integrative model to identify mRNA-miRNA associations in HCC.

### miRNA expression profiling

Aliquots of the same RNA samples used for the mRNA-seq analysis described above were analyzed by miRNA-seq. Indexed sequencing libraries were prepared from 200 ng total RNA using the QIAseq miRNA Library Kit (Qiagen) in the GESR according to the manufacturer’s instructions. Sequencing was performed on the NextSeq 550 System (Illumina) using 1 × 75 bp paired-end (SE75) read mode. The miRNA-seq data generated were then analyzed using the QIAseq miRNA quantification data analysis software. After quality assessment of the samples, FASTQ files were uploaded to the data analysis center at Qiagen for further analysis. In the first step, the unique molecular index (UMI) counts were calculated and primary miRNA mapping was performed. In the second step, the UMI counts were analyzed to calculate the changes in miRNA expression. The quantified data were then normalized using TMM method.

### Evaluation of dependence between tumor and adjacent normal tissues

We investigated the dependence between the tumor and adjacent normal tissue pairs across all miRNA-seq and mRNA-seq data. A correlation analysis between the tumor and adjacent normal tissues revealed that 87% of the correlations for the mRNAs and 70% of the correlations for the miRNAs have magnitudes lower than 0.3. In addition, a comparison of fixed effects and mixed effects models using AIC and BIC suggested that including random effects for individuals to account for the matching is not necessary. Thus, in our formulation of hierarchical integrative model, we assumed that the sample groups were independent.

### Hierarchical integrative model (HIM)

**Fig. 1:**
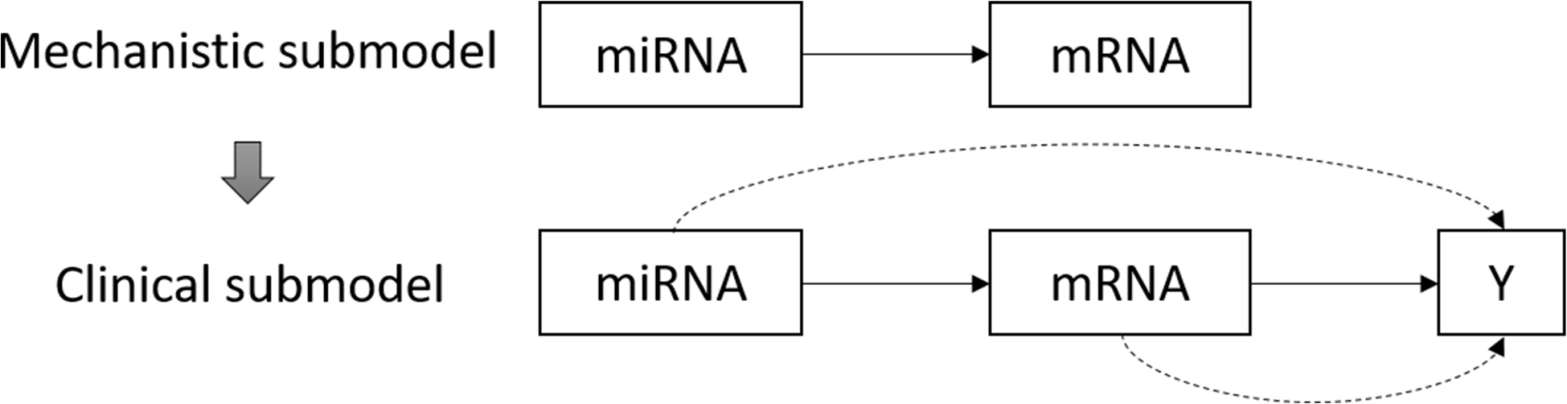
A hierarchical integrative model consisting of a mechanistic submodel and a clinical submodel

Hierarchical integrative model offers the opportunity to associate molecules measured in multiple omic studies across several levels to uncover novel relationships pertaining to disease status [15]. The HIM model we formulated for analysis of mRNA-seq and miRNA-seq data can be specified with two levels: (1) a mechanistic submodel relating mRNA to miRNA markers, and (2) a clinical submodel relating disease status to mRNA and miRNA, while accounting for the mechanistic relationships in the first level (Fig. 1).

**Fig. 2:**
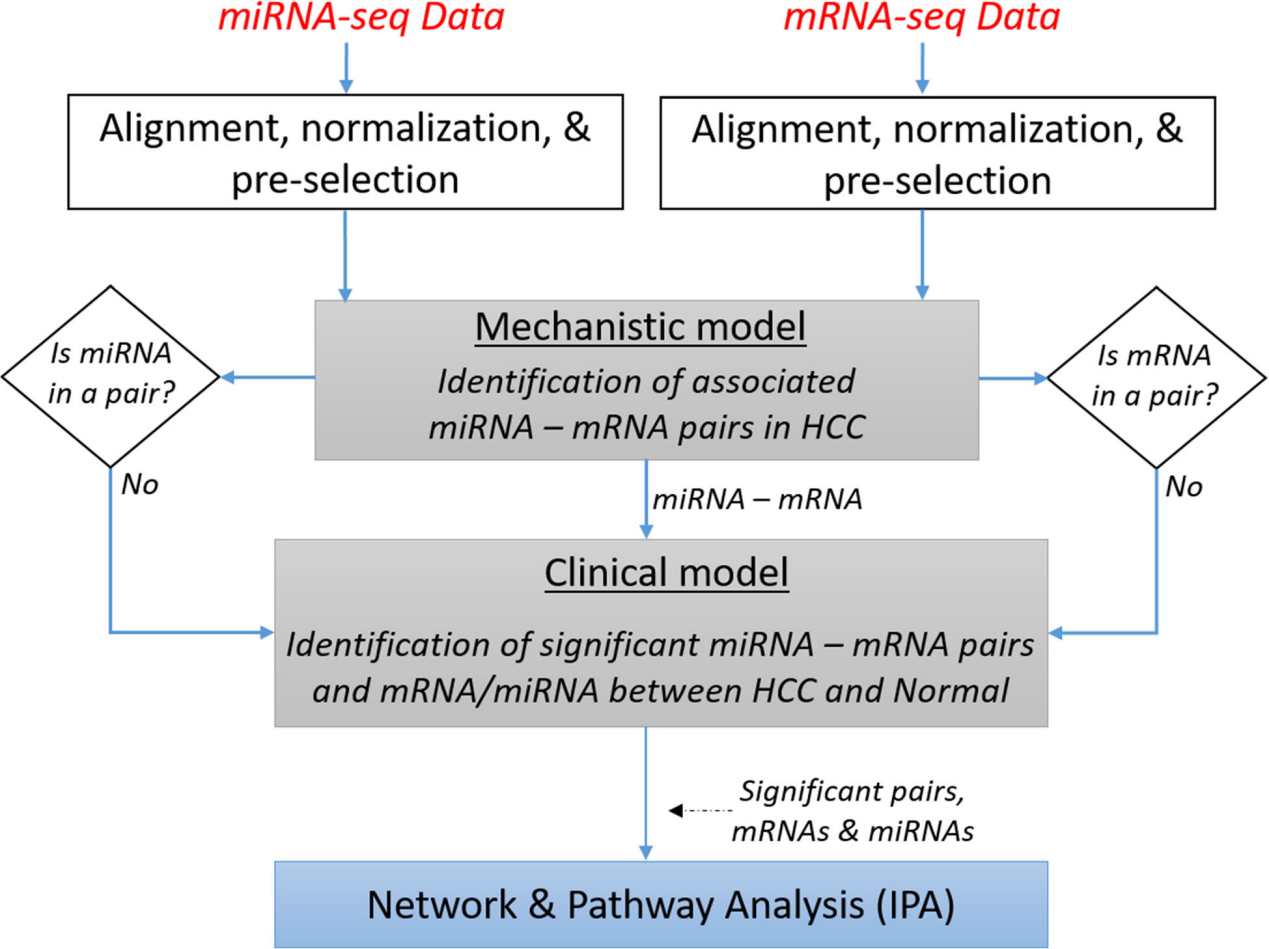
Overview of HIM to select miRNAs, mRNAs, and miRNA-mRNA pairs associated to disease

The mRNAs and miRNAs that go into the HIM are pre-selected using paired t-test considering only those mRNAs and miRNAs associated to the binary disease status Y (tumor or normal liver tissue). The mechanistic submodel fits multivariable models relating each mRNA to miRNAs. This is accomplished via lasso-regularized linear models using the R package glmnet [16]. The clinical submodel uses a penalized logistic regression model to relate the disease status Y to the linear predictors from the mechanistic submodel as well as mRNA and miRNA levels that were not selected in the mechanistic submodel. Figure 2 presents the steps involved in using HIM to identify miRNA-mRNA pairs associated to HCC.

### Mechanistic submodel

The mechanistic submodel is formulated as in Eq. (1), in which *η*_*j*_ corresponds to the linear predictor from the penalized regression model, relating mRNA_*j*_ to its associated miRNAs and e_*j*_ is the residual.

1 $$ {mRNA}_j=\underset{\cdot {\eta}_j}{\underbrace{\alpha_0+{\alpha}_1{miRNA}_{j1}+{\alpha}_2{miRNA}_{j2}+\cdots +{\alpha}_p{miRNA}_{jp}}}+{e}_j $$

### Clinical submodel

The clinical submodel is formulated as in Eq. (2), in which $\overline{mRNA}$ corresponds to the set of mRNAs that passed the t-test (FDR < 0.05) but were not picked in the mechanistic submodel (i.e., mRNAs related to Y but that do not have miRNAs associated with them); $\overline{miRNA}$ corresponds to the set of miRNAs that passed the t-test (FDR < 0.05) but were not picked in the mechanistic submodel (i.e., miRNAs not related to mRNAs but that could potentially affect the response Y). The logistic regression model in the clinical submodel is fit using a lasso penalty to identify relevant markers.

2 $$ \mathrm{logit}\ P\left(Y=1\right)={\beta}_0+\sum {\beta}_{\eta j}{\eta}_{j.}+\sum {\beta}_{ej}{e}_j+\sum {\beta}_k\overline{mRNA_k}+\sum {\beta}_l\overline{miRNA_l} $$

The selection of *η*_*j*._ would imply that the effects of mRNA_*j*_ on the outcome are modulated by the miRNAs associated to it, while the selection of *e*_*j*_ would imply that the effects of mRNA_*j*_ on the outcome result from factors other than those accounted for by its associated miRNAs. With this formulation, if *η*_*j*._ is selected, mRNA_j_ and all its associated miRNAs would be picked, although some of the miRNAs may not be relevant.

## Results

### Analysis of GU dataset by HIM

Prior to performing statistical analysis, the distribution of the data was evaluated. The mRNA and miRNA expression levels were then log-transformed to render their distributions fairly symmetric. We then used a paired t-test to identify miRNAs and mRNAs that are differentially expressed between the HCC and adjacent normal tissues. Adjusted *p*-values were calculated using Benjamini-Hochberg (BH) method. Fold changes were calculated considering the median of raw intensity values. Table 3 represents the number of features detected and the number of miRNAs and mRNAs selected with FDR < 0.05.

**Table 3:** Number of mRNAs, miRNAs, and miRNA-mRNA pairs selected by HIM using the GU dataset

		Statistical Analysis		Mechanistic Model	Clinical Model
	# of detected features	# of selected features with FDR < 0.05	total # of pairs	selected pairs (#mRNA) (#miRNA)	selected pairs (#mRNA) (#miRNA)
miRNA	2195	238	1,368,738	26,080 (3632) (238)	157 (19) (90)
mRNA	20,354	5751			

The miRNAs with FDR < 0.05 were matched to target pairs from databases using the miRNA target filter function that provides biological effects of miRNAs based on experimentally validated interactions from TarBase and miRecords, as well as predicted miRNA-mRNA interactions from TargetScan. The miRNA targets were then overlapped with the list of significant mRNAs from the mRNA-seq analysis to select reported targets of the significant miRNAs. A pathway analysis of this filtered set performed using IPA identified 469 pathways as significantly enriched with *p* < 0.05. FXR/RXR Activation, LXR/RXR Activation, Acute Phase Response Signaling, Hepatic Fibrosis / Hepatic Stellate Cell Activation, Sirtuin Signaling Pathway, and Valine Degradation I were selected as the top pathways enriched using the significant mRNAs.

**Fig. 3:**
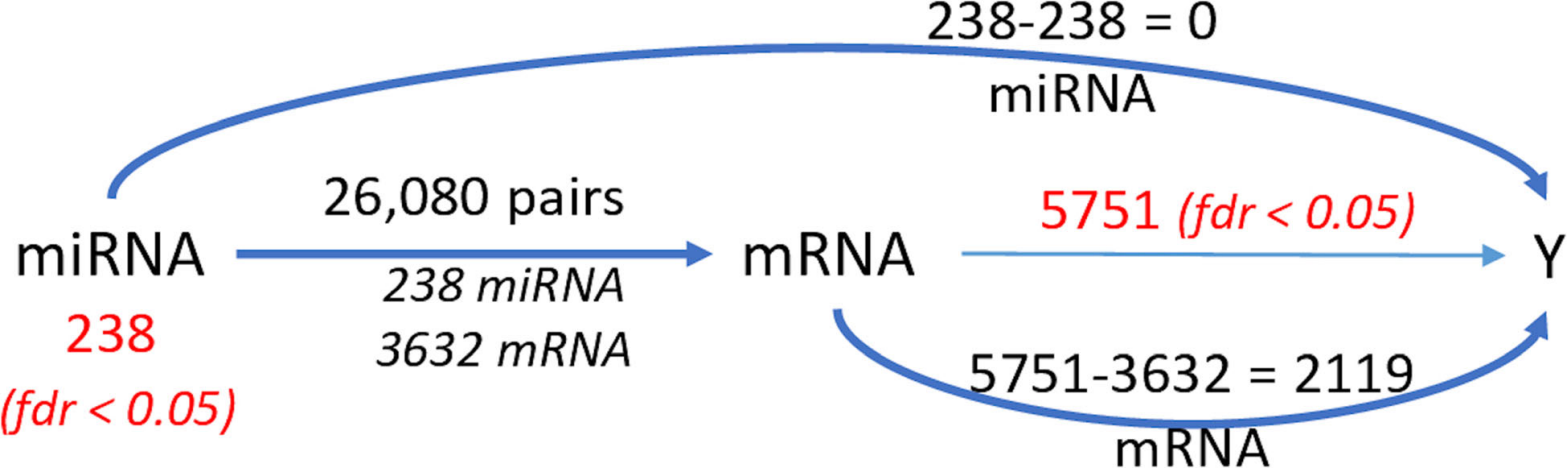
Number of mRNAs, miRNAs, and miRNA-mRNA pairs in the HIM for the GU dataset

The mechanistic submodel of HIM relating each of the significant (FDR < 0.05) 5751 mRNAs to 238 miRNAs using glmnet identified 26,080 miRNA-mRNA associations. All of the 238 miRNAs were involved in a potential pair with 3632 mRNAs as a result of the mechanistic submodel. All of the pairs and significant independent molecules that passed the univariate t-tests with FDR control were included in the clinical submodel for subsequent analysis. The clinical submodel selected 157 miRNA-mRNA pairs associated to HCC, which consist of 19 mRNAs and their related 90 miRNAs. Figure 3 depicts the number of molecules that were selected at each level of the analysis in the HIM. The selected 157 miRNA-mRNA pairs are included in the Supplementary Figure 1.

**Fig. 4:**
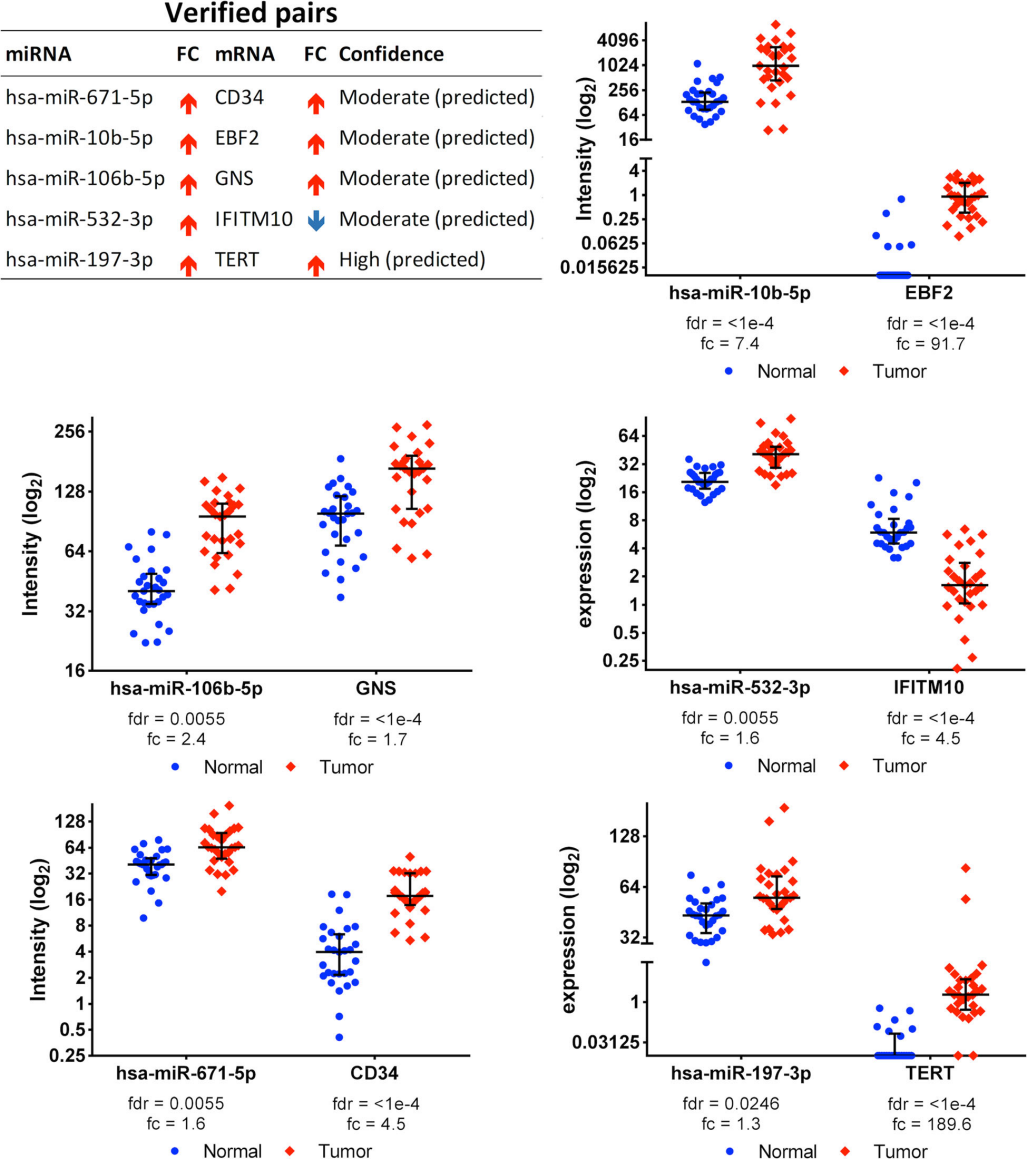
List and dotplots of the verified miRNA-mRNA pairs from the GU dataset

MicroRNA target filter analysis was then performed in IPA to match associated pairs that are reported or already verified experimentally. Of the 157 significant pairs selected by HIM, there are 40 experimentally verified pairs involving one of the molecules selected in the pair. Of these, five of the miRNA-mRNA pairs are exact matched pairs. Figure 4 presents these pairs with their confidence of prediction from the database and the dotplots of each pair.

**Fig. 5:**
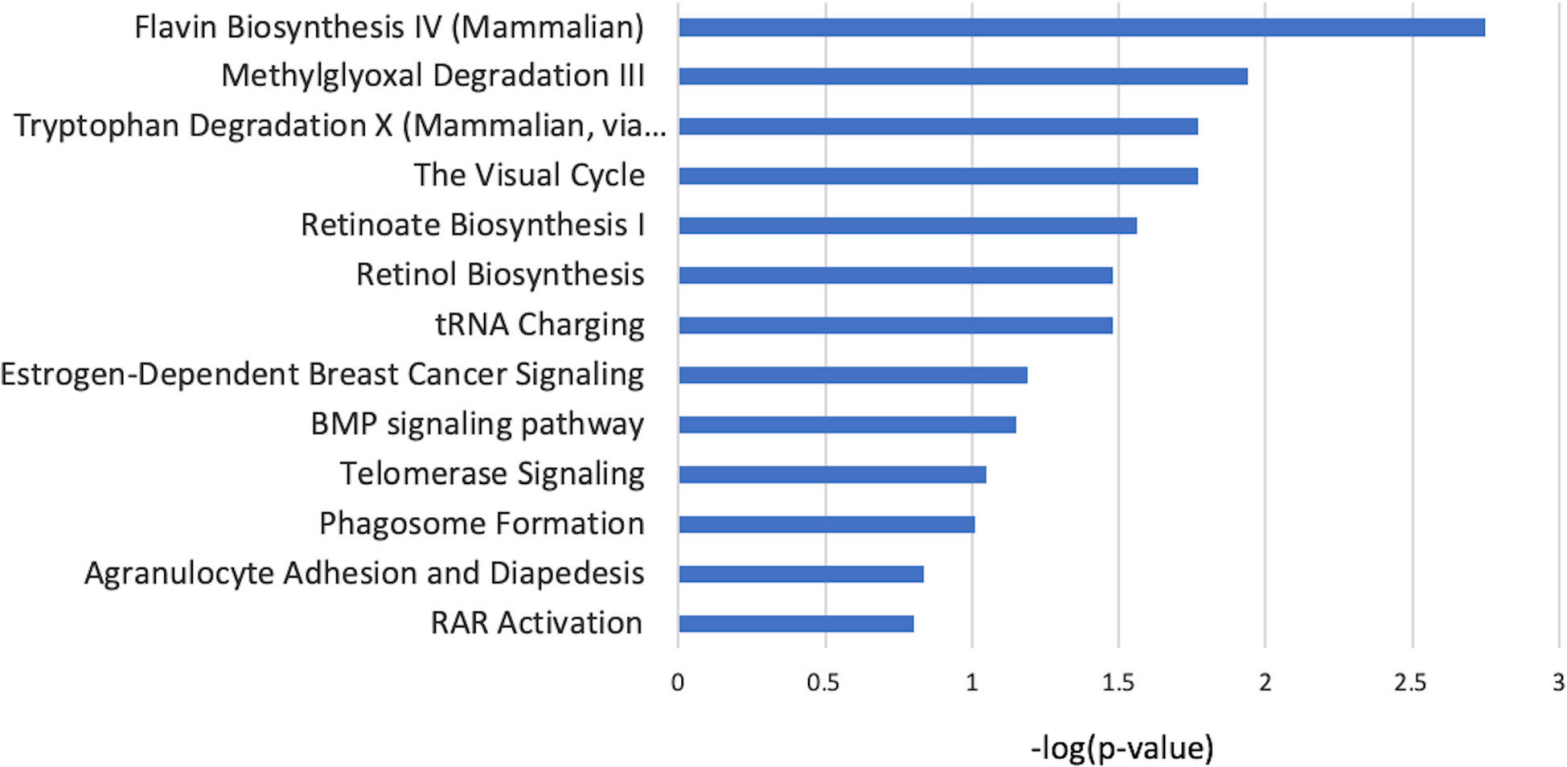
Pathways selected by IPA using the list of mRNAs and miRNAs from HIM based on the TCGA dataset

The miRNA-mRNA associations identified by HIM were analyzed in IPA to generate association networks from the knowledge-base and to further select the pathways that are dysregulated in HCC involving these miRNA-mRNA pairs. The significant pathways selected by the molecules involved in these pairs can be found in Fig. 5. A network generated using IPA showing the interactions and biological relevance of these selected molecules is provided in Supplementary Figure 2. The top biological processes depicted by this network are glomerular injury, inflammatory disease, and inflammatory response. The molecules drawn into this network from the database reflect the role of the selected molecules in pathogenesis of HCC as relevant to changes initiating inflammation, organismal injury, and connective tissue abnormalities.

### Analysis of TCGA dataset by HIM

**Fig. 6:**
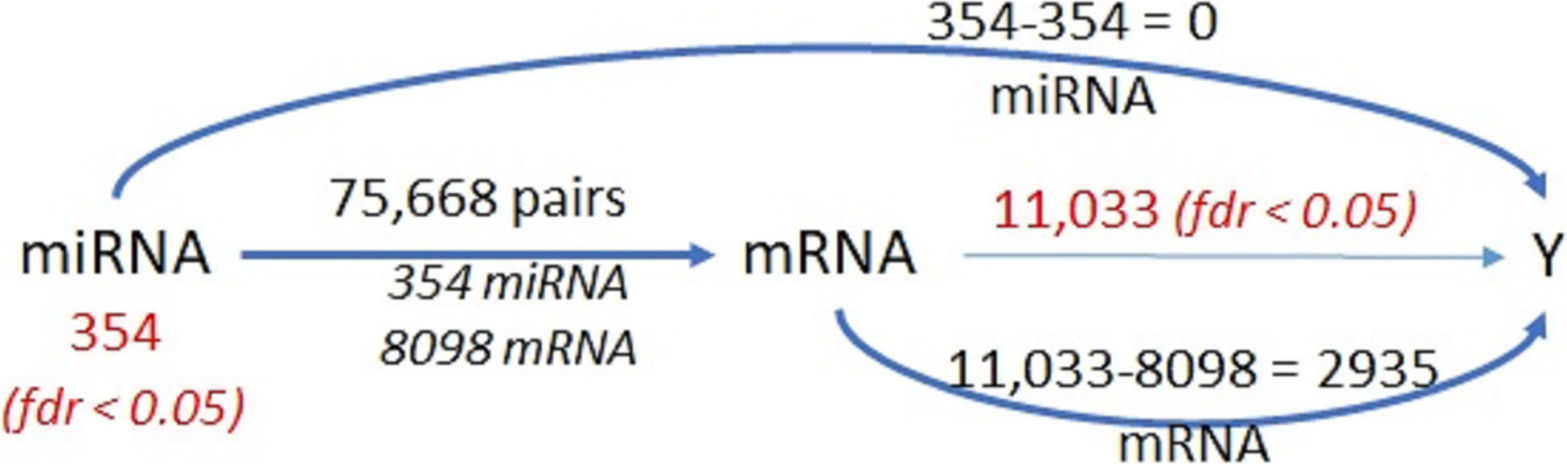
Number of mRNAs, miRNAs, and miRNA-mRNA pairs in the HIM for the TCGA dataset

We analyzed the TCGA dataset following the same approach as the GU dataset. Although miRNA-seq data consisted of 2588 miRNAs, there were more that 90% missing values for the 49 subjects considered and thus only 740 miRNAs were analyzed in this study. As shown in Table 4, there were 354 miRNAs and 11,033 mRNAs with FDR < 0.05 related to tumor vs. normal tissue. Figure 6 depicts the number of molecules that were selected at each level of the analysis in the HIM. A pathway analysis of significant miRNAs and mRNAs performed using IPA identified 522 pathways as significantly enriched with *p* < 0.05. Of these, 85% of the pathways overlap with the 469 significantly enriched pathways from GU dataset. Axonal Guidance Signaling, LXR/RXR Activation, LPS/IL-1 Mediated Inhibition of RXR Function, Role of Macrophages, Fibroblasts and Endothelial Cells in Rheumatoid Arthritis, Hepatic Fibrosis / Hepatic Stellate Cell Activation, FXR/RXR Activation, were selected as the top pathways enriched using the significant mRNAs.

**Table 4:** Number of molecules and miRNA-mRNA associations selected by HIM using the TCGA dataset

				Mechanistic Model	Clinical Model
	# of detected features	# of selected features with FDR < 0.05	total # of pairs	selected pairs (#mRNA) (#miRNA)	selected pairs (#mRNA) (#miRNA)
miRNA	740	354	3,905,682	75,668 (8098) (354)	263 (14) (136)
mRNA	18,407	11,033

Of the 263 significant miRNA-mRNA pairs selected by the HIM, there are 61 experimentally verified pairs involving one of the molecules selected in the pair, of which six pairs are exact match verified based on microRNA Target Filter analysis in IPA. Similar to the GU dataset, the pairs selected from the TCGA dataset overlap in regulation and function. The 263 miRNA-mRNA pairs and IPA generated network of interactions of these selected molecules are shown in Supplementary Figures 3 and 4, respectively. Significant pathways with *p*-value < 0.05, analyzed by IPA using the list of mRNAs and miRNAs from the hierarchical integrative model can be found in Supplementary Figure 5. The list and dot plots of the six verified miRNA-mRNA pairs are shown in Supplementary Figure 6.

While the GU dataset consists of 2195 miRNAs, only 740 miRNAs (about 1/3 of the miRNAs in the GU dataset) were considered in the TCGA dataset due to a large number of missing values in the TCGA data. Of these, 706 miRNAs overlapped between the GU and TCGA datasets. At the mechanistic submodel of the HIM, 1190 miRNA-mRNA pairs overlapped between the GU and TCGA datasets. Of these, 13 pairs have been reported as experimentally verified. Following this, the clinical model selected 157 pairs in the GU dataset consisting of 19 mRNAs and 90 miRNAs. Similarly, 263 pairs were selected in the TCGA dataset consisting of 14 mRNAs and 136 miRNAs. Although no overlapping exact pairs between the GU and TCGA datasets were found at this level, 26 of the miRNAs selected in the pairs overlapped between the two datasets, but each miRNA was paired to a different mRNA. Also, pathway analysis based on the selected mRNAs and the targets of the miRNAs revealed an overlap of 85% of the significant pathways between the two datasets. From this, we infer that the mRNAs selected in a pair and the targets of the miRNAs selected from each dataset may share the same pathway.

### Correlation analysis of GU and TCGA datasets

To compare the performance of HIM in selecting miRNA-mRNA pairs with more traditional approaches such as correlation analysis, we used a regularized generalized canonical correlation analysis (rgCCA), a component-based approach that aims to study the relationships between several sets of variables [17]. This analysis selected 36,963 miRNA-mRNA pairs (|r| > 0.5 and FDR < 0.05) from GU dataset and 36,962 miRNA-mRNA pairs (|r| > 0.5 and FDR < 0.05) from the TCGA dataset. MicroRNA target filter analysis in IPA revealed 983 and 258 verified exact pairs in the GU and TCGA datasets, respectively. Out of these, 20 and 65 miRNA-mRNA pairs in the GU and TCGA dataset, respectively, overlap with the pairs that were selected by HIM. Comparing the miRNA-mRNAs selected by HIM at the mechanistic submodel with the pairs selected by rgCCA, we found 104 experimentally verified pairs overlapped in the GU dataset. Similarly, 35 experimentally verified pairs overlapped between sets of pairs selected by rgCCA and HIM in the TCGA dataset. This shows that rgCCA and the mechanistic submodel of HIM tend to identify a large number of overlapping verified pairs. However, HIM offers the ability to discover miRNA-mRNA pairs related to the disease status, thus providing a more focused set of disease-associated pairs for further validation.

## Discussion

Recently discovered significance of miRNAs in homeostasis and disease have increased the importance of incorporating miRNAs into gene regulatory networks. HIMs offer the opportunity to associate molecules measured in multiple omic studies across several levels to discover novel relationships pertaining to disease status. There are several experimentally validated dual-upregulated as well as dual down-regulated miRNA-mRNA pairs found in IPA and in the literature. MiRNAs are thought to post-transcriptionally repress gene expression; however, the full extent of the mechanisms by which this occurs are not fully understood (i.e., repression of a repressor leading to dual upregulation, etc.) [18–20]. Several studies have demonstrated the ability of miRNAs to activate gene expression in response to various conditions in specific cell-types [10, 21, 22]. The purpose of this paper is to integrate miRNA-seq and mRNA-seq data to select associated miRNA-mRNA pairs that are of biological relevance to HCC.

### Dysregulation of miRNA-mRNA pairs in HCC

A literature search was conducted to validate selected molecules, many of which were found to be extensively reported in relation to HCC. Several of these selected molecules are already identified as being either diagnostic (VSP45, TERT, EBF2, hsa-miR-324-5p, hsa-miR-130a-3p, hsa-miR-200a-3p, hsa-miR-106b-5p, hsa-21-5p, hsa-424-5p), prognostic (CAP2, TRMT6, MT1JP, hsa-miR-10b-5p, hsa-miR-139-3p, hsa-miR-101, hsa-miR-122-5p, hsa-miR-1285-3p, hsa-miR-203a-3p), recurrence-related (CD34, hsa-miR-183), or therapeutic (AKR1B10, SLC25A4, CYP2B7, FBXL18, hsa-miR-146a-5p, hsa-miR-34a, hsa-miR-122, hsa-miR-671-5p) biomarker candidates for HCC [23–27]. These molecules appear to be involved in molecular events relating to inflammation, tumor microenvironment, cell cycle, apoptosis, and metabolism. These events in combination with key oncocytic molecular events facilitate pathogenesis, progression, and metastasis of HCC. This is evident by literature search findings as well as our pathway and network analysis results.

### MARCO as a potential target gene of hsa-miR-10b-5p

**Fig. 7:**
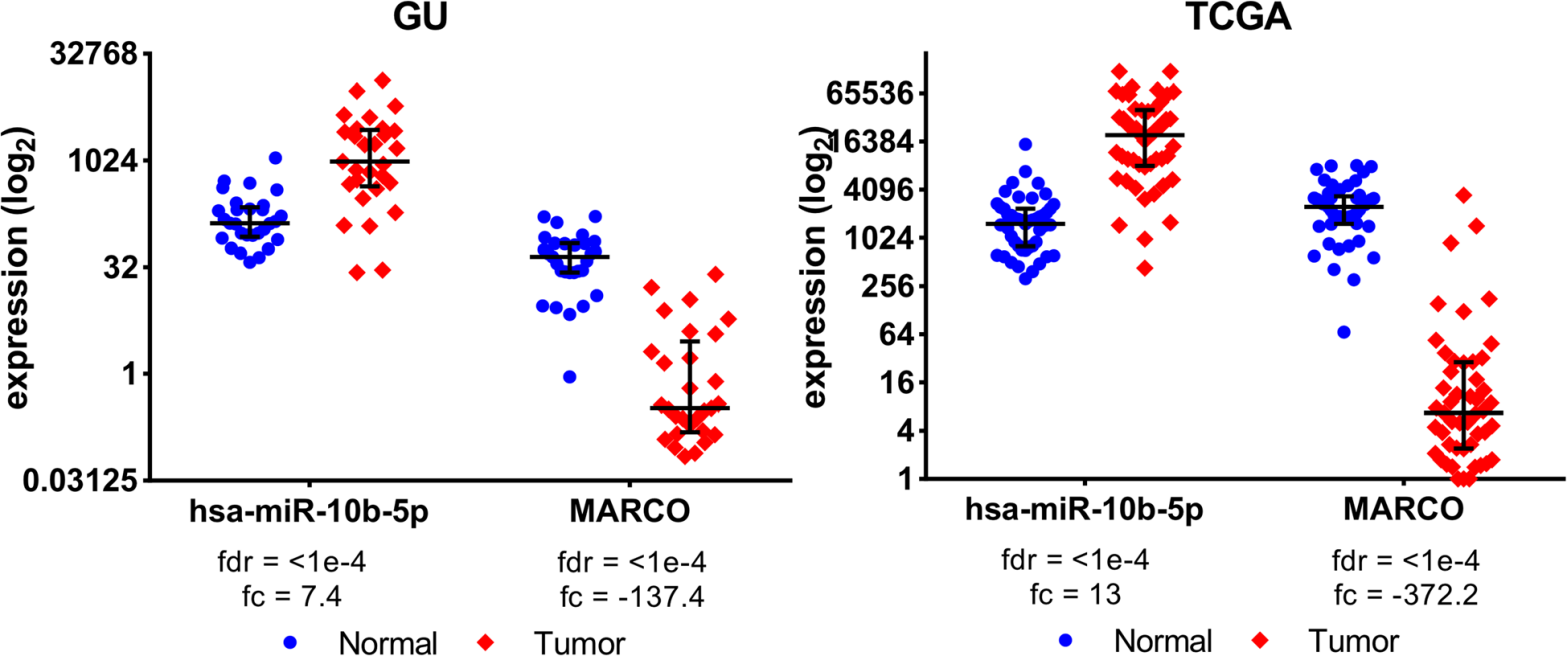
Dotplot of hsa-miR-10b-5p and MARCO in the GU and TCGA datasets

Our hierarchical integrated model results indicate that, macrophage scavenger receptor (MARCO) is associated with hsa-miR-10b-5p in both our GU and the TCGA datasets. Figure 7 shows the dot plots of the pairs selected from both datasets. The hsa-miR-10b-5p to MARCO pair is involved in HCC through increasing cellular proliferation, differentiation, and migration in HCC. According to miRecords, there are five experimentally verified targets of hsa-miR-10b, and MARCO has not been reported as a target. Decreased expression of MARCO is associated with poor prognosis in HCC, potentially due to its role in modulating inflammatory signaling [28]. Recently, MARCO has been identified as a marker of non-inflammatory macrophages in the liver [29]. Expression of MARCO has been used to define a population of suppressive tumor-associated macrophages. Thus, upregulation of MARCO in HCC suggests a phenotype of heightened immunosuppression. Interestingly, hsa-miR-10b was also found to be downregulated in macrophages and explored as a circulating biomarker of HCC recurrence in bone marrow cells, suggesting a possible link between hsa-miR-10b and MARCO [30]. Hsa-miR-10b has also been identified as a prognostic biomarker of HCC in a study that used 33 miRNA signatures to characterize HCC using TCGA data [26]. Overexpression of miR-10b in HCC has been reported to promote cell proliferation, migration and invasion [31]. Thus, MARCO can be a potential target of hsa-miR-10b that is worth verifying. Further, additional miRNAs were identified by our analysis as having an influence on MARCO expression. These include hsa-miR-125b-2-3p, hsa-miR-146a-5p, hsa-miR-200a-3p, and hsa-miR-221 that are all known diagnostic biomarkers of HCC.

### miRNA-mRNA pairs involved in tumor inflammation

Pairs involving regulation of MARCO, IFITM10, and CD34 appear to function in HCC through changes involving tumor microenvironment and inflammation. IFITM10 is an interferon-induced transmembrane protein known to play a role in anti-viral response in HCC caused by virally-induced etiology [32]. IFITM10 was found to be targeted by hsa-miR-106b-5p in our analysis, a miRNA known to activate immunoregulatory T cells and myeloid-derived suppressor cells [33]. Hsa-miR-146a-5p is thought to be down-regulated in HCC leading to immune suppression and HCC-induced NK cell dysfunction. MARCO is a target gene found to be regulated by hsa-miR-146a-5p in our analysis suggesting functional relevance [34]. Hsa-miR-122 and Has-miR-146a-5p are both thought to be involved in regulating STAT3 signaling, a key pathway involved in immune suppression in tumor microenvironment [34]. Hsa-miR-122 is the most abundant miRNA in healthy human liver and down-regulation in HCC is associated with poor prognosis [25]. Further, restoration of miR-122 levels is used to indicate therapeutic response and sensitivity with several drugs used to treat HCC. Similarly, hsa-miR-34a suppression in HCC has been linked to increased CCL22 levels known to recruit regulatory T cells involved in immune escape [35]. Lastly, CD34 expression on liver sinusoidal endothelial cells is linked to chronic liver inflammation and thought to play a role in the pathogenesis of HCC from underlying cirrhosis [36]. The role of miRNA-mRNA regulation in HCC could prove useful to assess the immune status of these tumors and predict response to these novel therapeutics.

### miRNA-mRNA pairs involving cell cycle and apoptosis

Pairs involving TRMT6, SLC25A40, MT1JP, and EBF2 are involved in HCC through regulation of cell cycle and apoptosis contributing to disease progression and metastasis [37, 38]. Hsa-miR-671-5p was associated with CD34 and EBF2 in our analysis and is known to be associated with metastasis, proliferation, and EMT in HCC [39]. Dysregulated expression of hsa-miR-7 and hsa-miR-532-3p in HCC leads to increased proliferation, invasion, and metastasis through the PI3K/AKT signaling pathway [40]. Further, hsa-miR-532-3p along with hsa-miR-139 are associated with CHRD, FLAD1, MT1JP, and EBF2 in our analysis and have been identified as tumor suppressors in HCC that inhibit cell proliferation, migration, and invasion [41]. MT1JP is also thought to act as a tumor suppressor through regulating a series of pathways involving p53, such as the cell cycle, apoptosis and proliferation [42]. Similarly, TRMT6 upregulation correlates with poor prognosis in HCC and is a MYC target gene involved in G2M checkpoint and cell-cycle regulation [43]. Hsa-miR-101-5p is another molecule regulated by MYC signaling that is repressed, leading to progression and metastasis of HCC [44]. Hsa-miR-200a is thought to suppress proliferation in HCC by induction of G1 arrest through CDK6. Similarly, has-miR-125b-2-3p is an upstream regulator of oncogenic sirtuin that also causes G1 arrest. Interestingly, Sirtuin signaling was one of the top pathways identified from this analysis.

### miRNA-mRNA pairs involved in metabolic regulation

Many miRNA-mRNA pairs identified contribute to aberrant metabolism in HCC and some of these are further associated with distinct etiology. For instance, target genes (FLAD1, SLFNL1, GNS, FBXL18, CYP2B7P) are commonly upregulated genes in metabolic-induced HCC and are associated with risk factors, such as diabetes, obesity, and other metabolic syndromes. These genes are commonly upregulated in a subclass of malignant HCC tumors under metabolic stress and associated with hypoxic behavior, epithelial-to-mesenchymal transition, and increased fatty acid biosynthesis [45]. CYP2B7P was found to be a target gene of hsa-miR-122 that is a diagnostic biomarker of HCC as well as of non-alcoholic fatty liver disease (NAFLD) [25]. MiR-122 is also known to affect metabolism of HCC cells through pyruvate kinase [46]. CYP2B7P was also found to be a gene target of hsa-miR-183-5p that is a biomarker of HCC and diabetes. Many of the top pathways identified from these selected miRNA-mRNA pairs are involved in metabolic regulation, including FXR/RXR activation, LPS-IL-1 mediated inhibition of RXR function, LXR/RXR activation, valine degradation, fatty acid B-oxidation, hepatic cholestasis and tryptophan degradation. Tryptophan degradation was one of the pathways found to overlap between significant pairs identified by TCGA and our datasets. In addition, we previously identified several molecules from the FXR/RXR activation pathway as being differentially expressed in HCC patients involved in bile acid homeostasis, lipid and glucose metabolism [47].

### Upstream miRNA regulators of TERT

Telomerase plays an important role in maintaining telomere length and chromosomal stability in hepatocytes that is often dysfunctional in cirrhotic liver and HCC [25]. Early-stage and virally-induced tumors are often associated with telomere dysfunction along with p53 mutations. The relevance of TERT and TP53 in these HCC cases can be visualized by the top network depicting interaction amongst our selected miRNA-mRNA pairs (Supplemental Figure 2). Over 68% of HCC cases are thought to have TERT mutations influencing telomere maintenance and susceptibility for viral-genome integration [48]. In addition, TERT is a commonly amplified gene in HCC patients and its presence has been used to characterize a subpopulation of HCC cases [49]. For example, HCC patients with HBV are more likely to have mutations in P53 than TERT because HBV often integrates at the location of the TERT gene. In contrast, patients with HCV and HCC patients of Black or African American race are more likely to have TERT promoter mutations than p53. A cluster of genes appears to be associated with this subpopulation, including CAP2 and CHRD, that are involved in BMP signaling [50]. BMP signaling was another pathway found to overlap between the selected pairs identified by TCGA and our datasets and is associated with this subpopulation. TERT was found to be regulated by several miRNAs including hsa-miR-21-5p, hsa-miR-130b-5p, hsa-miR-675-3p, and hsa-miR-488-3p. All of these miRNAs are thought to play a role in HCC [49]. This makes miRNA pairs involved with regulating TERT a significant focus worth additional validation.

### Hierarchical integrative model to identify miRNA-mRNA pairs

Some of the miRNA-mRNA pairs identified in this study are reported to have involvement in inflammation, cell cycle regulation, apoptosis, and metabolism. In addition to the functional categories that most pairs are involved in, several pairs are involved in resistance mechanisms, splicing alterations, and epigenetic dysregulation. Several known pairs stemming from the selected 19 mRNAs and associated 90 miRNAs have been experimentally validated in the database with different partners. For instance, TRMT6 is a known target gene of both miR-21 and miR-146a-5p. From our analysis, miR-21 and miR-221 were both selected as being involved in significant pairs functioning in HCC. These two miRNAs are known oncomiRs in HCC whose mRNA targets are often found to act as tumor suppressors influencing tumor proliferation and migration [25]. In addition to TRMT6, several other genes were identified as targets of miR-21 that have not yet been validated, including TERT and TPS45. However, we recognize that the actual role the above mentioned pairs may have in the pathogenesis of HCC is much more complex and not fully known. The ability of HIMs to identify novel miRNA-mRNA pairs that have not been validated in existing databases is a strength that can be used for discovery of novel targets. For example, our analysis identified four genes that are potential targets of miR-221 (MARCO, AKR1B10, CD34, and LARS) that could have similar importance in HCC. This shows the ability of HIMs to predict novel miRNA-mRNA pairs of biological significance in relation to HCC that warrant additional validation in the future.

## Conclusion

In this study, we used hierarchical integrative model to integrate miRNA-seq and mRNA-seq data acquired by analysis of paired tumor and adjacent normal liver tissues from the same set of patients with HCC. The model led to identification of key miRNA-mRNA pairs and associated pathways that are potentially involved in HCC pathogenesis. The results strongly support the hypothesis that miRNAs are important regulators of mRNA expressions in HCC. Furthermore, these results emphasize the biological relevance of studying miRNA-mRNA pairs when analyzing miRNA-seq and mRNA-seq datasets. As mRNA-miRNA regulation generally exists in various biological systems, understanding the functionality of mRNA-miRNA is critical for elucidating its underlying role in disease pathology. Combining regulators of disease across multiple -omics levels can aid in providing a more synergistic view of disease progression in general and a roadmap to addressing the imbalance between statistically and biologically relevant information.

## Supplementary information

**Additional file 1: Figure S1.** miRNA-mRNA pairs identified using HIM for GU dataset. An upward arrow denotes a positive fold change and a downward arrow denotes a negative fold change when comparing HCC vs normal.

**Additional file 2: Figure S2.** miRNA-mRNA interaction network for GU dataset. The network was constructed based on mRNAs and miRNAs selected by the HIM from the GU dataset. The molecules drawn into this network reflect the role of the selected molecules in pathogenesis of HCC. The molecules highlighted in red are up-regulated and those in blue are down-regulated in HCC. The network was generated through the use of IPA (QIAGEN Inc., https://www.qiagenbioinformatics.com/products/ingenuity-pathway-analysis).

**Additional file 3: Figure S3.** miRNA-mRNA pairs identified using HIM for TCGA dataset. An upward arrow denotes a positive fold change and a downward arrow denotes a negative fold change when comparing HCC vs normal.

**Additional file 4: Figure S4.** miRNA-mRNA interaction network for TCGA dataset. The network was constructed based on mRNAs and miRNAs selected by the HIM from the TCGA dataset. The molecules highlighted in red are up-regulated and those in blue are down-regulated in HCC. The network was generated through the use of IPA (QIAGEN Inc., https://www.qiagenbioinformatics.com/products/ingenuity-pathway-analysis).

**Additional file 5: Figure S5.** Top canonical pathways enriched in TCGA dataset. The significant pathways were selected with *p*-value < 0.05 analyzed by IPA using the list of mRNAs and miRNAs selected from the hierarchical integrative model based on the TCGA dataset.

**Additional file 6: Figure S6.** Verified miRNA-mRNA pairs from the TCGA dataset. The list shown consists of verified miRNA-mRNA pairs with their confidence in target prediction from IPA target filter analysis. The expression of the miRNA and their predicted target is shown as dotplot.

## Data Availability

R code implementation of HIM and pre-processed miRNA-seq and mRNA-seq data are available at https://github.com/ressomlab/HIM. The TCGA dataset were obtained from the TCGA Research Network (https://portal.gdc.cancer.gov/projects/TCGA-LIHC; Project ID- TCGA-LIHC; dbGaP Study Accession: phs000178), using the Broad Institute firehose download link, http://gdac.broadinstitute.org/ (accessed on January 20, 2019). Level 3 mRNA-seq and miRNA-seq data were obtained. The clinical information was available from the NCI genomic data commons data portal at (https://portal.gdc.cancer.gov/projects/TCGA-LIHC).

## Abbreviations

AA: African American
Adj-N: Adjacent Normal Tissue
AFP: Alpha-Fetoprotein
ALT: Alanine Aminotransferase
AST: Aspartate Aminotransferase
BMI: Body Mass Index
CNV: Copy Number Variation
EA: European American
EM: Expectation Maximization
FDR: False Discovery Rate
GESR: Genomics and Epigenomics Shared Resource
GU: Georgetown University
GUMC: Georgetown University Medical Center
HBV: Hepatitis B virus
HCC: Hepatocellular Carcinoma
HCV: Hepatitis C Virus
HIM: Hierarchical Integrative Model
IQR: Inter Quartile Range
IPA: Ingenuity Pathway Analysis
MELD: Model For End-Stage Liver Disease
MGUH: Medstar Georgetown University Hospital
rgCCA: regularized generalized Canonical Correlation Analysis
SD: Standard deviation
STAR: Spliced Transcripts Alignment to a Reference
TCGA: The Cancer Genome Atlas
UMI: Unique Molecular Index
