# Supplementary material for: Identification of miRNA-mRNA associations in hepatocellular carcinoma using hierarchical integrative model
Source: BMC Med Genomics. 2020 Mar 30;13:56. doi: 10.1186/s12920-020-0706-1 (PMC7106691; doi:10.1186/s12920-020-0706-1)
Supplement: Supplementary file 1 — Additional file 1: Figure S1. miRNA-mRNA pairs identified using HIM for GU dataset. An upward arrow denotes a positive fold change and a downward arrow denotes a negative fold change when comparing HCC vs normal. [file 12920_2020_706_MOESM1_ESM.pdf]

miRNA - mRNA pair				miRNA - mRNA pair				miRNA - mRNA pair				miRNA - mRNA pair			
hsa-miR-130a-3p	↓	↑	AKR1B10	hsa-miR-1285-3p	↑	↑	CD34	hsa-miR-182-5p	↑	↑	VPS45	hsa-miR-34c-5p	↑	↓	CHRD
hsa-miR-4686	↓	↑	CAP2	hsa-miR-221-3p	↑	↑	CD34	hsa-miR-190b	↑	↑	VPS45	hsa-miR-589-5p	↑	↓	CHRD
hsa-miR-187-3p	↓	↑	CD34	hsa-miR-3144-3p	↑	↑	CD34	hsa-miR-21-5p	↑	↑	VPS45	hsa-miR-671-3p	↑	↓	CHRD
hsa-miR-4686	↓	↑	CD34	hsa-miR-671-5p	↑	↑	CD34	hsa-miR-3144-3p	↑	↑	VPS45	hsa-miR-15b-3p	↑	↓	CYP2B7P
hsa-miR-675-3p	↓	↑	CD34	hsa-miR-10b-5p	↑	↑	EBF2	hsa-miR-324-5p	↑	↑	VPS45	hsa-miR-183-5p	↑	↓	CYP2B7P
hsa-miR-1231	↓	↑	EBF2	hsa-miR-1285-3p	↑	↑	EBF2	hsa-miR-34c-3p	↑	↑	VPS45	hsa-miR-196b-5p	↑	↓	CYP2B7P
hsa-miR-136-3p	↓	↑	EBF2	hsa-miR-532-3p	↑	↑	EBF2	hsa-miR-452-5p	↑	↑	VPS45	hsa-miR-301a-5p	↑	↓	CYP2B7P
hsa-miR-139-3p	↓	↑	EBF2	hsa-miR-589-5p	↑	↑	EBF2	hsa-miR-454-3p	↑	↑	VPS45	hsa-miR-532-3p	↑	↓	CYP2B7P
hsa-miR-4686	↓	↑	EBF2	hsa-miR-671-5p	↑	↑	EBF2	hsa-miR-5010-3p	↑	↑	VPS45	hsa-miR-589-5p	↑	↓	CYP2B7P
hsa-miR-511-5p	↓	↑	EBF2	hsa-miR-15b-3p	↑	↑	FLAD1	hsa-miR-93-5p	↑	↑	VPS45	hsa-miR-106b-5p	↑	↓	IFITM10
hsa-miR-597-5p	↓	↑	EBF2	hsa-miR-589-5p	↑	↑	FLAD1	hsa-miR-95-5p	↑	↑	VPS45	hsa-miR-532-3p	↑	↓	IFITM10
hsa-miR-4686	↓	↑	FBXL18	hsa-miR-1180-3p	↑	↑	GNS	hsa-miR-96-5p	↑	↑	VPS45	hsa-miR-10b-5p	↑	↓	MARCO
hsa-miR-139-3p	↓	↑	FLAD1	hsa-miR-34a-5p	↑	↑	GNS	hsa-miR-139-3p	↓	↓	CHRD	hsa-miR-130b-5p	↑	↓	MARCO
hsa-miR-19a-5p	↓	↑	FLAD1	hsa-miR-34b-5p	↑	↑	GNS	hsa-miR-4686	↓	↓	CHRD	hsa-miR-151a-3p	↑	↓	MARCO
hsa-miR-383-5p	↓	↑	FLAD1	hsa-miR-34c-3p	↑	↑	GNS	hsa-miR-548aq-3p	↓	↓	CHRD	hsa-miR-221-3p	↑	↓	MARCO
hsa-miR-424-5p	↓	↑	FLAD1	hsa-miR-4454	↑	↑	GNS	hsa-miR-122-3p	↓	↓	CYP2B7P	hsa-miR-3662	↑	↓	MARCO
hsa-miR-675-3p	↓	↑	FLAD1	hsa-miR-548l	↑	↑	GNS	hsa-miR-203a-3p	↓	↓	CYP2B7P	hsa-miR-3684	↑	↓	MARCO
hsa-miR-1208	↓	↑	GNS	hsa-miR-671-3p	↑	↑	GNS	hsa-miR-378c	↓	↓	CYP2B7P	hsa-miR-454-3p	↑	↓	MARCO
hsa-miR-19a-5p	↓	↑	GNS	hsa-miR-7-5p	↑	↑	GNS	hsa-miR-4686	↓	↓	CYP2B7P	hsa-miR-4659a-3p	↑	↓	MARCO
hsa-miR-200b-3p	↓	↑	GNS	hsa-miR-221-3p	↑	↑	LARS	hsa-miR-378d	↓	↓	IFITM10	hsa-miR-532-3p	↑	↓	MARCO
hsa-miR-130a-3p	↓	↑	LARS	hsa-miR-339-3p	↑	↑	LARS	hsa-miR-424-5p	↓	↓	IFITM10	hsa-miR-589-3p	↑	↓	MARCO
hsa-miR-4686	↓	↑	MGC27345	hsa-miR-34c-3p	↑	↑	LARS	hsa-miR-101-5p	↓	↓	MARCO	hsa-miR-887-5p	↑	↓	MARCO
hsa-miR-19a-5p	↓	↑	SLC25A40	hsa-miR-190b	↑	↑	SLC25A40	hsa-miR-125b-2-3p	↓	↓	MARCO	hsa-miR-942-5p	↑	↓	MARCO
hsa-miR-1208	↓	↑	TERT	hsa-miR-3144-3p	↑	↑	SLC25A40	hsa-miR-146a-5p	↓	↓	MARCO	hsa-miR-34a-3p	↑	↓	MT1JP
hsa-miR-19a-5p	↓	↑	TERT	hsa-miR-339-3p	↑	↑	SLC25A40	hsa-miR-200a-3p	↓	↓	MARCO	hsa-miR-34b-3p	↑	↓	MT1JP
hsa-miR-383-5p	↓	↑	TERT	hsa-miR-34c-3p	↑	↑	SLC25A40	hsa-miR-219b-5p	↓	↓	MARCO	hsa-miR-34c-3p	↑	↓	MT1JP
hsa-miR-488-3p	↓	↑	TERT	hsa-miR-4742-3p	↑	↑	SLC25A40	hsa-miR-378d	↓	↓	MARCO	hsa-miR-3684	↑	↓	MT1JP
hsa-miR-6503-3p	↓	↑	TERT	hsa-miR-5010-3p	↑	↑	SLC25A40	hsa-miR-6503-3p	↓	↓	MARCO	hsa-miR-423-3p	↑	↓	MT1JP
hsa-miR-6719-3p	↓	↑	TERT	hsa-miR-1306-5p	↑	↑	TERT	hsa-miR-8077	↓	↓	MARCO	hsa-miR-423-5p	↑	↓	MT1JP
hsa-miR-675-3p	↓	↑	TERT	hsa-miR-130b-5p	↑	↑	TERT	hsa-miR-122-5p	↓	↓	MT1JP	hsa-miR-4791	↑	↓	MT1JP
hsa-miR-8077	↓	↑	TERT	hsa-miR-15b-3p	↑	↑	TERT	hsa-miR-1231	↓	↓	MT1JP	hsa-miR-95-5p	↑	↓	MT1JP
hsa-miR-4686	↓	↑	TRMT6	hsa-miR-190b	↑	↑	TERT	hsa-miR-136-3p	↓	↓	MT1JP	hsa-miR-2277-5p	↑	↓	SLFNL1
hsa-miR-146b-3p	↓	↑	VPS45	hsa-miR-197-3p	↑	↑	TERT	hsa-miR-139-5p	↓	↓	MT1JP	hsa-miR-4742-3p	↑	↓	SLFNL1
hsa-miR-4472	↓	↑	VPS45	hsa-miR-21-5p	↑	↑	TERT	hsa-miR-146b-3p	↓	↓	MT1JP	hsa-miR-671-3p	↑	↓	SLFNL1
hsa-miR-4721	↓	↑	VPS45	hsa-miR-3144-3p	↑	↑	TERT	hsa-miR-4484	↓	↓	MT1JP				
hsa-miR-675-3p	↓	↑	VPS45	hsa-miR-3613-5p	↑	↑	TERT	hsa-miR-548aq-3p	↓	↓	MT1JP				
hsa-miR-7704	↓	↑	VPS45	hsa-miR-548l	↑	↑	TERT	hsa-miR-6503-3p	↓	↓	MT1JP				
hsa-miR-130b-3p	↑	↑	AKR1B10	hsa-miR-570-3p	↑	↑	TERT	hsa-miR-6719-3p	↓	↓	MT1JP				
hsa-miR-188-5p	↑	↑	AKR1B10	hsa-miR-887-5p	↑	↑	TERT	hsa-miR-130a-3p	↓	↓	SLFNL1				
hsa-miR-221-3p	↑	↑	AKR1B10	hsa-miR-106b-5p	↑	↑	VPS45	hsa-miR-30e-5p	↓	↓	SLFNL1				
hsa-miR-10b-5p	↑	↑	CD34	hsa-miR-15b-5p	↑	↑	VPS45	hsa-miR-339-3p	↑	↓	CHRD				

Up-regulation	↑
Down-regulation	↓
