# Supplementary figures and images for: Identification of miRNA-mRNA associations in hepatocellular carcinoma using hierarchical integrative model
Source: BMC Med Genomics. 2020 Mar 30;13:56. doi: 10.1186/s12920-020-0706-1 (PMC7106691; doi:10.1186/s12920-020-0706-1)

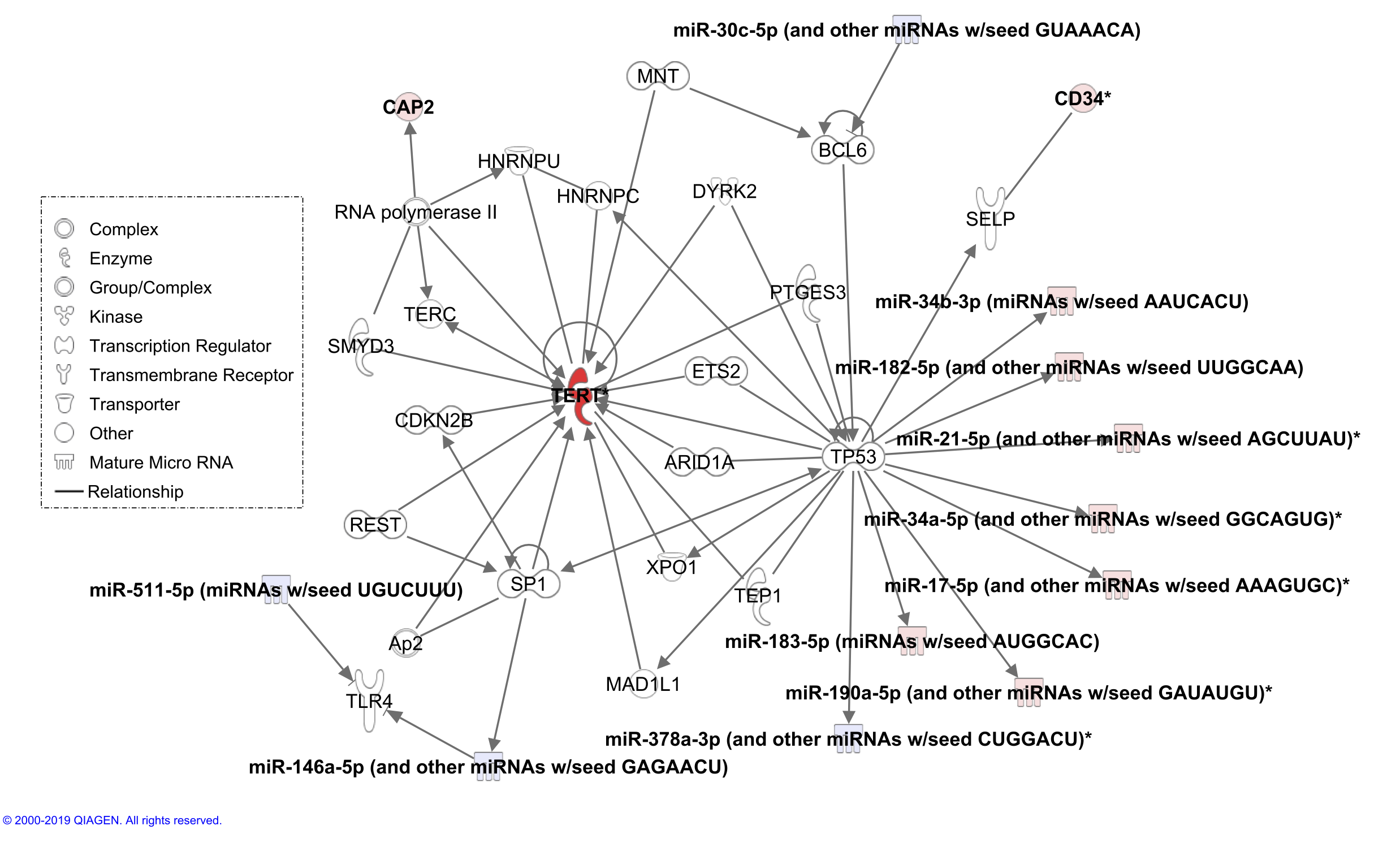

Supplement: Supplementary file 2 — Additional file 2: Figure S2. miRNA-mRNA interaction network for GU dataset. The network was constructed based on mRNAs and miRNAs selected by the HIM from the GU dataset. The molecules drawn into this network reflect the role of the selected molecules in pathogenesis of HCC. The molecules highlighted in red are up-regulated and those in blue are down-regulated in HCC. The network was generated through the use of IPA (QIAGEN Inc., https://www.qiagenbioinformatics.com/products/ingenuity-pathway-analysis). [file 12920_2020_706_MOESM2_ESM.png]

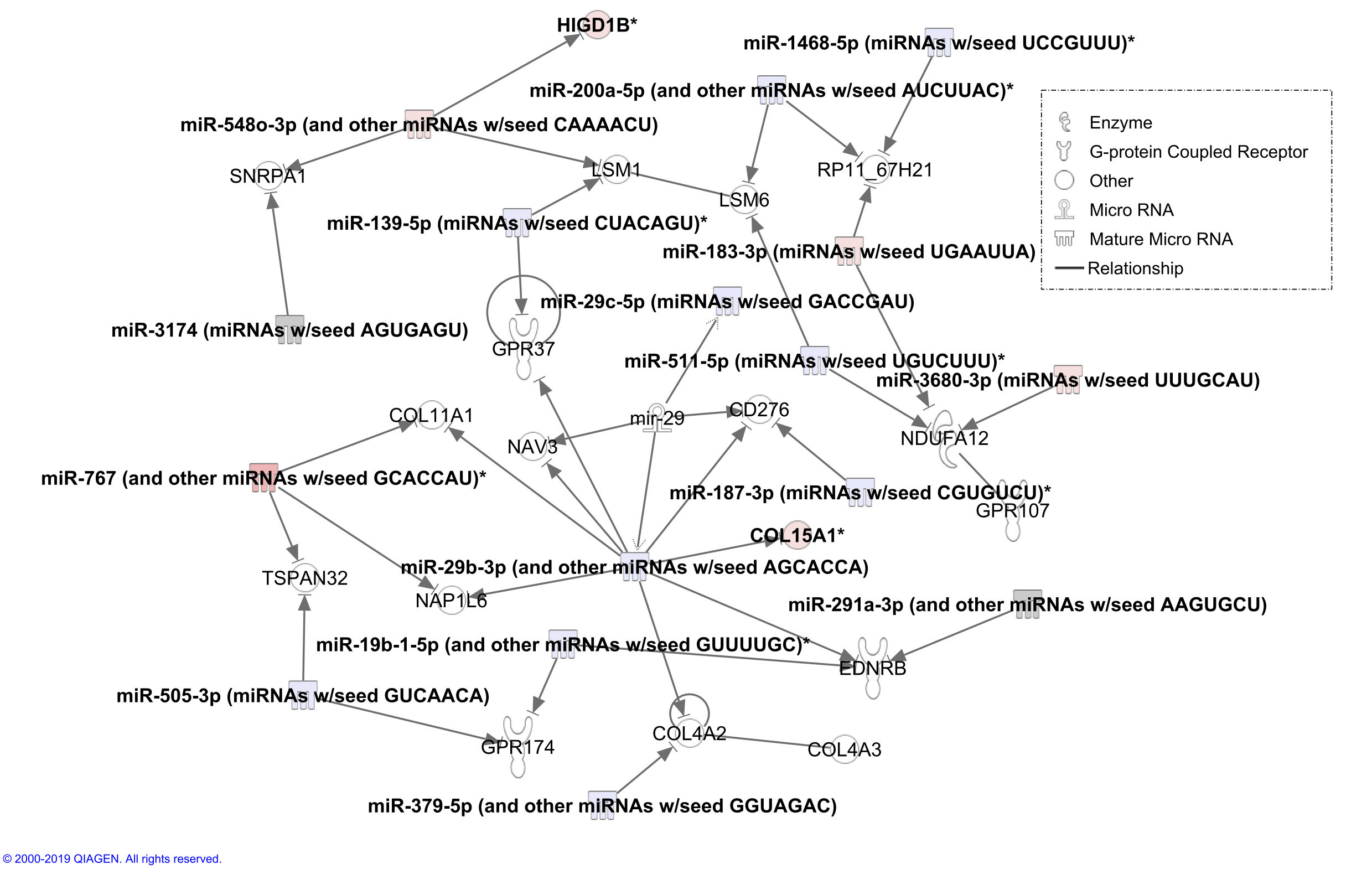

Supplement: Supplementary file 4 — Additional file 4: Figure S4. miRNA-mRNA interaction network for TCGA dataset. The network was constructed based on mRNAs and miRNAs selected by the HIM from the TCGA dataset. The molecules highlighted in red are up-regulated and those in blue are down-regulated in HCC. The network was generated through the use of IPA (QIAGEN Inc., https://www.qiagenbioinformatics.com/products/ingenuity-pathway-analysis). [file 12920_2020_706_MOESM4_ESM.png]

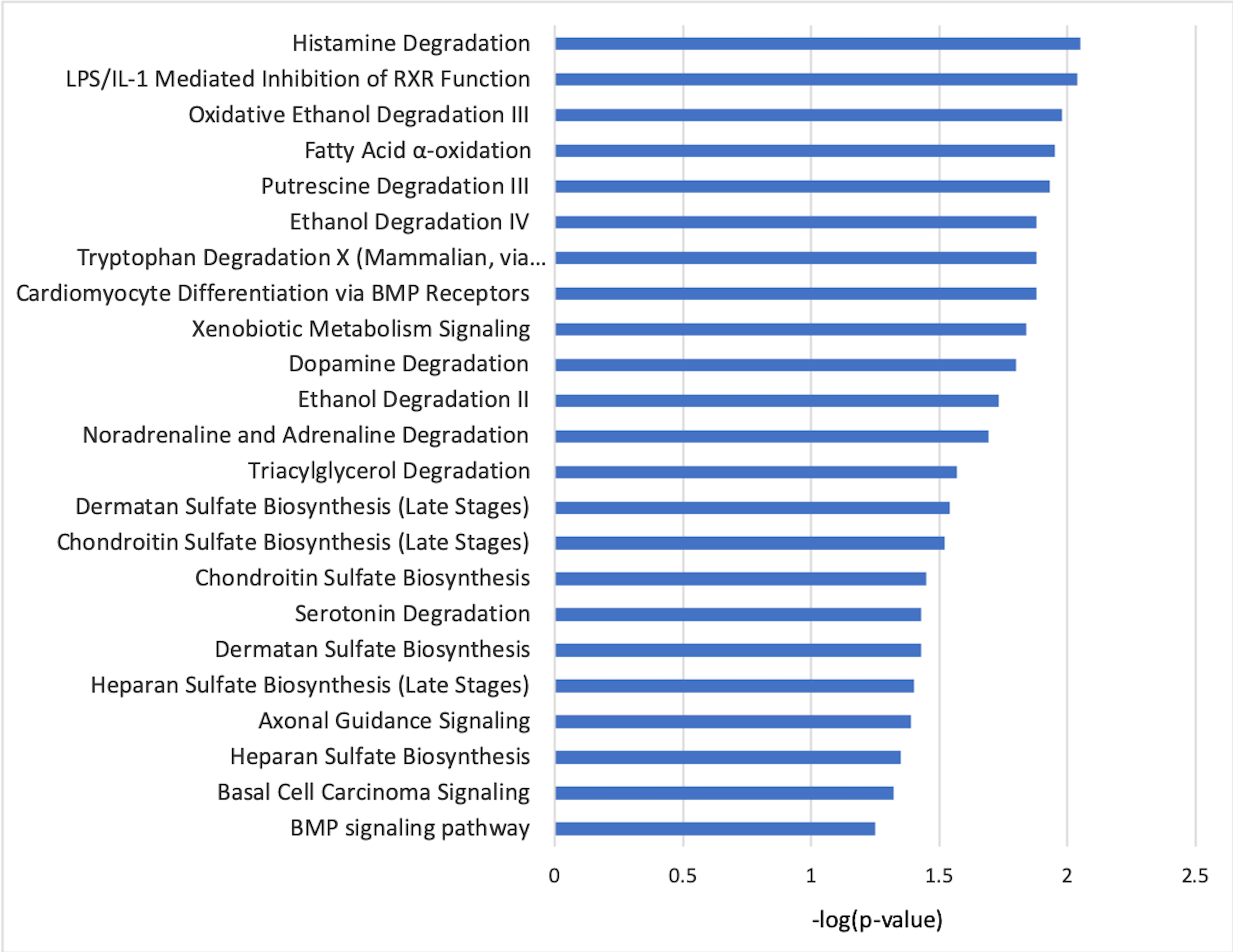

Supplement: Supplementary file 5 — Additional file 5: Figure S5. Top canonical pathways enriched in TCGA dataset. The significant pathways were selected with p-value < 0.05 analyzed by IPA using the list of mRNAs and miRNAs selected from the hierarchical integrative model based on the TCGA dataset. [file 12920_2020_706_MOESM5_ESM.png]

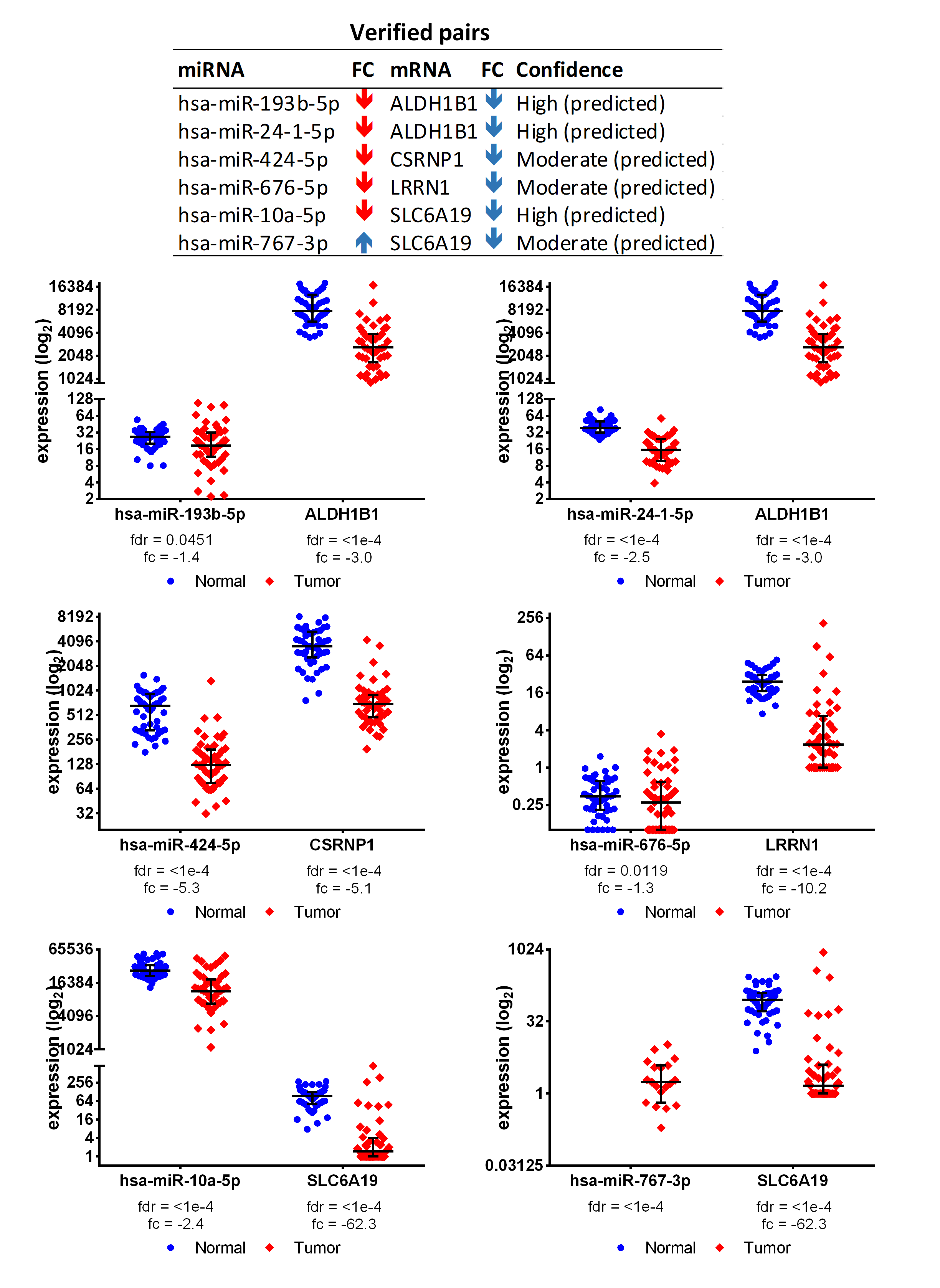

Supplement: Supplementary file 6 — Additional file 6: Figure S6. Verified miRNA-mRNA pairs from the TCGA dataset. The list shown consists of verified miRNA-mRNA pairs with their confidence in target prediction from IPA target filter analysis. The expression of the miRNA and their predicted target is shown as dotplot. [file 12920_2020_706_MOESM6_ESM.png]
