# Supplementary material for: Identification of miRNA-mRNA associations in hepatocellular carcinoma using hierarchical integrative model
Source: BMC Med Genomics. 2020 Mar 30;13:56. doi: 10.1186/s12920-020-0706-1 (PMC7106691; doi:10.1186/s12920-020-0706-1)
Supplement: Supplementary file 3 — Additional file 3: Figure S3. miRNA-mRNA pairs identified using HIM for TCGA dataset. An upward arrow denotes a positive fold change and a downward arrow denotes a negative fold change when comparing HCC vs normal. [file 12920_2020_706_MOESM3_ESM.pdf]

miRNA - mRNA pair				miRNA - mRNA pair				miRNA - mRNA pair				miRNA - mRNA pair			
hsa-miR-26a-5p	↓	↑	PCGF1	hsa-miR-502-3p	↑	↑	COL15A1	hsa-miR-1976	↓	↓	NDST3	hsa-miR-665	↓	↓	SLC6A19
hsa-miR-101-3p	↓	↑	PCGF1	hsa-miR-767-5p	↑	↑	COL15A1	hsa-miR-148a-3p	↓	↓	AGBL4	hsa-miR-1247-5p	↓	↓	SLC6A19
hsa-miR-10a-5p	↓	↑	PCGF1	hsa-miR-767-3p	↑	↑	COL15A1	hsa-miR-30c-5p	↓	↓	AGBL4	hsa-miR-3648	↓	↓	SLC6A19
hsa-miR-142-5p	↓	↑	PCGF1	hsa-miR-1254	↑	↑	COL15A1	hsa-miR-30c-2-3p	↓	↓	AGBL4	hsa-miR-3944-3p	↓	↓	SLC6A19
hsa-miR-144-5p	↓	↑	PCGF1	hsa-miR-10b-5p	↑	↑	TOMM40L	hsa-miR-203a-3p	↓	↓	AGBL4	hsa-miR-151a-3p	↑	↓	LRRN1
hsa-miR-193a-5p	↓	↑	PCGF1	hsa-miR-188-5p	↑	↑	TOMM40L	hsa-miR-200b-5p	↓	↓	AGBL4	hsa-miR-518b	↑	↓	LRRN1
hsa-miR-200a-5p	↓	↑	PCGF1	hsa-miR-532-5p	↑	↑	TOMM40L	hsa-miR-30e-3p	↓	↓	AGBL4	hsa-miR-517-5p	↑	↓	LRRN1
hsa-miR-30e-3p	↓	↑	PCGF1	hsa-miR-1180-3p	↑	↑	TOMM40L	hsa-miR-505-3p	↓	↓	AGBL4	hsa-miR-25-3p	↑	↓	ALDH1B1
hsa-miR-379-5p	↓	↑	PCGF1	hsa-miR-10b-5p	↑	↑	SPTAN1	hsa-miR-802	↓	↓	AGBL4	hsa-miR-132-3p	↑	↓	ALDH1B1
hsa-miR-490-5p	↓	↑	PCGF1	hsa-miR-34b-3p	↑	↑	SPTAN1	hsa-miR-378c	↓	↓	AGBL4	hsa-miR-99b-3p	↑	↓	ALDH1B1
hsa-miR-505-5p	↓	↑	PCGF1	hsa-miR-99b-3p	↑	↑	SPTAN1	hsa-miR-3648	↓	↓	AGBL4	hsa-miR-361-5p	↑	↓	ALDH1B1
hsa-miR-592	↓	↑	PCGF1	hsa-miR-151a-3p	↑	↑	SPTAN1	hsa-miR-3687	↓	↓	AGBL4	hsa-miR-330-5p	↑	↓	ALDH1B1
hsa-miR-3944-3p	↓	↑	PCGF1	hsa-miR-508-5p	↑	↑	SPTAN1	hsa-miR-21-3p	↓	↓	CSRNP1	hsa-miR-425-5p	↑	↓	ALDH1B1
hsa-miR-19b-1-5p	↓	↑	COL15A1	hsa-miR-589-5p	↑	↑	SPTAN1	hsa-miR-139-3p	↓	↓	CSRNP1	hsa-miR-767-5p	↑	↓	ALDH1B1
hsa-miR-192-5p	↓	↑	COL15A1	hsa-miR-891a-5p	↑	↑	SPTAN1	hsa-miR-7-1-3p	↓	↓	CSRNP1	hsa-miR-760	↑	↓	ALDH1B1
hsa-miR-148a-3p	↓	↑	COL15A1	hsa-miR-301b-3p	↑	↑	SPTAN1	hsa-miR-223-3p	↓	↓	CSRNP1	hsa-miR-1278	↑	↓	ALDH1B1
hsa-miR-139-5p	↓	↑	COL15A1	hsa-miR-3144-5p	↑	↑	SPTAN1	hsa-miR-30b-5p	↓	↓	CSRNP1	hsa-miR-3144-3p	↑	↓	ALDH1B1
hsa-miR-187-3p	↓	↑	COL15A1	hsa-miR-3174	↑	↑	SPTAN1	hsa-miR-30e-3p	↓	↓	CSRNP1	hsa-miR-3074-3p	↑	↓	ALDH1B1
hsa-miR-210-3p	↓	↑	COL15A1	hsa-miR-3677-3p	↑	↑	SPTAN1	hsa-miR-424-5p	↓	↓	CSRNP1	hsa-miR-3168	↑	↓	ALDH1B1
hsa-miR-144-3p	↓	↑	COL15A1	hsa-miR-3680-3p	↑	↑	SPTAN1	hsa-miR-486-5p	↓	↓	CSRNP1	hsa-miR-10b-5p	↑	↓	NDST3
hsa-miR-30e-5p	↓	↑	COL15A1	hsa-miR-10b-5p	↑	↑	HIGD1B	hsa-miR-489-3p	↓	↓	CSRNP1	hsa-miR-188-5p	↑	↓	NDST3
hsa-miR-383-5p	↓	↑	COL15A1	hsa-miR-132-3p	↑	↑	HIGD1B	hsa-miR-490-5p	↓	↓	CSRNP1	hsa-miR-362-3p	↑	↓	NDST3
hsa-miR-486-5p	↓	↑	COL15A1	hsa-miR-151a-3p	↑	↑	HIGD1B	hsa-miR-450b-5p	↓	↓	CSRNP1	hsa-miR-151a-3p	↑	↓	NDST3
hsa-miR-511-5p	↓	↑	COL15A1	hsa-miR-502-3p	↑	↑	HIGD1B	hsa-miR-139-5p	↓	↓	ADAMTS13	hsa-miR-581	↑	↓	NDST3
hsa-miR-33b-5p	↓	↑	COL15A1	hsa-miR-1276	↑	↑	HIGD1B	hsa-miR-200b-3p	↓	↓	ADAMTS13	hsa-miR-767-3p	↑	↓	NDST3
hsa-miR-1468-5p	↓	↑	COL15A1	hsa-miR-30c-5p	↓	↓	LRRN1	hsa-miR-130a-3p	↓	↓	ADAMTS13	hsa-miR-132-3p	↑	↓	AGBL4
hsa-miR-675-3p	↓	↑	COL15A1	hsa-miR-30c-2-3p	↓	↓	LRRN1	hsa-miR-144-5p	↓	↓	ADAMTS13	hsa-miR-34c-5p	↑	↓	AGBL4
hsa-miR-378c	↓	↑	COL15A1	hsa-miR-10a-5p	↓	↓	LRRN1	hsa-miR-424-5p	↓	↓	ADAMTS13	hsa-miR-589-5p	↑	↓	AGBL4
hsa-miR-3614-3p	↓	↑	COL15A1	hsa-miR-200b-3p	↓	↓	LRRN1	hsa-miR-490-5p	↓	↓	ADAMTS13	hsa-miR-550a-5p	↑	↓	AGBL4
hsa-miR-3664-5p	↓	↑	COL15A1	hsa-miR-490-3p	↓	↓	LRRN1	hsa-miR-490-3p	↓	↓	ADAMTS13	hsa-miR-548o-3p	↑	↓	AGBL4
hsa-miR-101-3p	↓	↑	TOMM40L	hsa-miR-450b-5p	↓	↓	LRRN1	hsa-miR-450b-5p	↓	↓	ADAMTS13	hsa-miR-1278	↑	↓	AGBL4
hsa-miR-30c-2-3p	↓	↑	TOMM40L	hsa-miR-1258	↓	↓	LRRN1	hsa-miR-3648	↓	↓	ADAMTS13	hsa-miR-7-2-3p	↑	↓	CSRNP1
hsa-miR-139-5p	↓	↑	TOMM40L	hsa-miR-3648	↓	↓	LRRN1	hsa-miR-31-3p	↓	↓	BMP10	hsa-miR-10b-5p	↑	↓	CSRNP1
hsa-miR-139-3p	↓	↑	TOMM40L	hsa-miR-676-5p	↓	↓	LRRN1	hsa-miR-101-3p	↓	↓	BMP10	hsa-miR-222-3p	↑	↓	CSRNP1
hsa-miR-130a-3p	↓	↑	TOMM40L	hsa-miR-22-3p	↓	↓	ALDH1B1	hsa-miR-139-3p	↓	↓	BMP10	hsa-miR-188-5p	↑	↓	CSRNP1
hsa-miR-450b-5p	↓	↑	TOMM40L	hsa-miR-23a-5p	↓	↓	ALDH1B1	hsa-miR-142-5p	↓	↓	BMP10	hsa-miR-99b-3p	↑	↓	CSRNP1
hsa-miR-19b-1-5p	↓	↑	SPTAN1	hsa-miR-24-1-5p	↓	↓	ALDH1B1	hsa-miR-142-3p	↓	↓	BMP10	hsa-miR-452-5p	↑	↓	CSRNP1
hsa-miR-26b-3p	↓	↑	SPTAN1	hsa-miR-187-3p	↓	↓	ALDH1B1	hsa-miR-144-5p	↓	↓	BMP10	hsa-miR-589-5p	↑	↓	CSRNP1
hsa-miR-192-3p	↓	↑	SPTAN1	hsa-let-7g-3p	↓	↓	ALDH1B1	hsa-miR-193a-5p	↓	↓	BMP10	hsa-miR-891a-5p	↑	↓	CSRNP1
hsa-miR-210-3p	↓	↑	SPTAN1	hsa-miR-122-5p	↓	↓	ALDH1B1	hsa-miR-30e-5p	↓	↓	BMP10	hsa-miR-934	↑	↓	CSRNP1
hsa-miR-200b-3p	↓	↑	SPTAN1	hsa-miR-122-3p	↓	↓	ALDH1B1	hsa-miR-424-5p	↓	↓	BMP10	hsa-miR-3677-3p	↑	↓	CSRNP1
hsa-miR-30b-5p	↓	↑	SPTAN1	hsa-miR-142-3p	↓	↓	ALDH1B1	hsa-miR-663a	↓	↓	BMP10	hsa-miR-10b-5p	↑	↓	ADAMTS13
hsa-miR-29c-5p	↓	↑	SPTAN1	hsa-miR-193a-5p	↓	↓	ALDH1B1	hsa-miR-1468-5p	↓	↓	BMP10	hsa-miR-188-5p	↑	↓	ADAMTS13
hsa-miR-29c-3p	↓	↑	SPTAN1	hsa-miR-194-3p	↓	↓	ALDH1B1	hsa-miR-450b-5p	↓	↓	BMP10	hsa-miR-330-5p	↑	↓	ADAMTS13
hsa-miR-574-3p	↓	↑	SPTAN1	hsa-miR-200a-5p	↓	↓	ALDH1B1	hsa-miR-101-5p	↓	↓	AQP7	hsa-miR-421	↑	↓	ADAMTS13
hsa-miR-33b-5p	↓	↑	SPTAN1	hsa-miR-455-3p	↓	↓	ALDH1B1	hsa-miR-139-5p	↓	↓	AQP7	hsa-miR-10b-5p	↑	↓	BMP10
hsa-miR-1468-5p	↓	↑	SPTAN1	hsa-miR-665	↓	↓	ALDH1B1	hsa-miR-490-3p	↓	↓	AQP7	hsa-miR-183-3p	↑	↓	BMP10
hsa-miR-24-1-5p	↓	↑	HIGD1B	hsa-miR-3614-3p	↓	↓	ALDH1B1	hsa-let-7b-5p	↓	↓	SLC6A19	hsa-miR-222-3p	↑	↓	BMP10
hsa-miR-101-3p	↓	↑	HIGD1B	hsa-miR-3664-5p	↓	↓	ALDH1B1	hsa-let-7b-3p	↓	↓	SLC6A19	hsa-miR-106b-3p	↑	↓	BMP10
hsa-miR-200b-3p	↓	↑	HIGD1B	hsa-miR-3944-3p	↓	↓	ALDH1B1	hsa-miR-19b-1-5p	↓	↓	SLC6A19	hsa-miR-34c-5p	↑	↓	BMP10
hsa-miR-144-5p	↓	↑	HIGD1B	hsa-miR-139-5p	↓	↓	NDST3	hsa-miR-139-3p	↓	↓	SLC6A19	hsa-miR-520a-3p	↑	↓	BMP10
hsa-miR-30e-3p	↓	↑	HIGD1B	hsa-miR-139-3p	↓	↓	NDST3	hsa-miR-10a-5p	↓	↓	SLC6A19	hsa-miR-767-3p	↑	↓	BMP10
hsa-miR-490-5p	↓	↑	HIGD1B	hsa-miR-142-3p	↓	↓	NDST3	hsa-miR-145-5p	↓	↓	SLC6A19	hsa-miR-765	↑	↓	BMP10
hsa-miR-490-3p	↓	↑	HIGD1B	hsa-miR-144-5p	↓	↓	NDST3	hsa-miR-145-3p	↓	↓	SLC6A19	hsa-miR-1180-3p	↑	↓	BMP10
hsa-miR-511-5p	↓	↑	HIGD1B	hsa-miR-194-3p	↓	↓	NDST3	hsa-miR-200a-5p	↓	↓	SLC6A19	hsa-miR-3616-3p	↑	↓	BMP10
hsa-miR-450b-5p	↓	↑	HIGD1B	hsa-miR-200a-5p	↓	↓	NDST3	hsa-miR-30e-3p	↓	↓	SLC6A19	hsa-miR-3168	↑	↓	AQP7
hsa-miR-34a-5p	↑	↑	PCGF1	hsa-miR-380-5p	↓	↓	NDST3	hsa-miR-363-3p	↓	↓	SLC6A19	hsa-miR-34a-5p	↑	↓	SLC6A19
hsa-miR-9-5p	↑	↑	PCGF1	hsa-miR-424-5p	↓	↓	NDST3	hsa-miR-383-5p	↓	↓	SLC6A19	hsa-miR-452-5p	↑	↓	SLC6A19
hsa-miR-873-5p	↑	↑	PCGF1	hsa-miR-424-3p	↓	↓	NDST3	hsa-miR-424-5p	↓	↓	SLC6A19	hsa-miR-517-5p	↑	↓	SLC6A19
hsa-miR-1180-3p	↑	↑	PCGF1	hsa-miR-490-5p	↓	↓	NDST3	hsa-miR-483-3p	↓	↓	SLC6A19	hsa-miR-502-3p	↑	↓	SLC6A19
hsa-miR-1278	↑	↑	PCGF1	hsa-miR-490-3p	↓	↓	NDST3	hsa-miR-490-3p	↓	↓	SLC6A19	hsa-miR-508-3p	↑	↓	SLC6A19
hsa-miR-10b-5p	↑	↑	COL15A1	hsa-miR-511-5p	↓	↓	NDST3	hsa-miR-494-3p	↓	↓	SLC6A19	hsa-miR-767-5p	↑	↓	SLC6A19
hsa-miR-106b-3p	↑	↑	COL15A1	hsa-miR-505-5p	↓	↓	NDST3	hsa-miR-193b-5p	↓	↓	SLC6A19	hsa-miR-1276	↑	↓	SLC6A19
hsa-miR-99b-3p	↑	↑	COL15A1	hsa-miR-1185-5p	↓	↓	NDST3	hsa-miR-505-5p	↓	↓	SLC6A19	hsa-miR-3144-5p	↑	↓	SLC6A19
hsa-miR-330-5p	↑	↑	COL15A1	hsa-miR-802	↓	↓	NDST3	hsa-miR-582-3p	↓	↓	SLC6A19	hsa-miR-3144-3p	↑	↓	SLC6A19
hsa-miR-339-3p	↑	↑	COL15A1	hsa-miR-450b-5p	↓	↓	NDST3	hsa-miR-744-3p	↓	↓	SLC6A19				
